# A non-printed integrated-circuit textile for wireless theranostics

**DOI:** 10.1038/s41467-021-25075-8

**Published:** 2021-08-12

**Authors:** Yuxin Yang, Xiaofei Wei, Nannan Zhang, Juanjuan Zheng, Xing Chen, Qian Wen, Xinxin Luo, Chong-Yew Lee, Xiaohong Liu, Xingcai Zhang, Jun Chen, Changyuan Tao, Wei Zhang, Xing Fan

**Affiliations:** 1 College of Chemistry and Chemical Engineering, Chongqing University, Chongqing, China; 2 Chongqing Institute of Green and Intelligent Technology, Chinese Academy of Sciences, Chongqing, China; 3 Industrial Technology Research Institute of Chongqing University, Chongqing, China; 4 John A. Paulson School of Engineering and Applied Sciences, Harvard University, Cambridge, MA USA; 5 School of Pharmaceutical Sciences, University Sains Malaysia, Penang, Malaysia; 6 School of Engineering, Massachusetts Institute of Technology, Cambridge, MA USA; 7 Department of Bioengineering, University of California, Los Angeles, Los Angeles, CA USA

**Keywords:** Sensors and biosensors, Biosensors

## Abstract

While the printed circuit board (PCB) has been widely considered as the building block of integrated electronics, the world is switching to pursue new ways of merging integrated electronic circuits with textiles to create flexible and wearable devices. Herein, as an alternative for PCB, we described a non-printed integrated-circuit textile (NIT) for biomedical and theranostic application via a weaving method. All the devices are built as fibers or interlaced nodes and woven into a deformable textile integrated circuit. Built on an electrochemical gating principle, the fiber-woven-type transistors exhibit superior bending or stretching robustness, and were woven as a textile logical computing module to distinguish different emergencies. A fiber-type sweat sensor was woven with strain and light sensors fibers for simultaneously monitoring body health and the environment. With a photo-rechargeable energy textile based on a detailed power consumption analysis, the woven circuit textile is completely self-powered and capable of both wireless biomedical monitoring and early warning. The NIT could be used as a 24/7 private AI “nurse” for routine healthcare, diabetes monitoring, or emergencies such as hypoglycemia, metabolic alkalosis, and even COVID-19 patient care, a potential future on-body AI hardware and possibly a forerunner to fabric-like computers.

## Abstract (web summary)

The typical approach to electronics is to integrate sensors, power units, and controlling components on a printed circuit board (PCB). Here, the authors demonstrate a self-powered and fully integrated combination of sensors and controlling components that is woven, rather than integrated onto a PCB, allowing for wearable health monitoring.”

## Introduction

With the rapid progress in flexible electronics, daily personal physiological data monitoring and analysis using artificial intelligence (AI) assisted healthcare can define a healthier and smarter lifestyle^1–6^. For instance, unusual symptoms of many life-threatening diseases can be obtained from daily sweating profiles, serving as an effective quantitative index for early diagnosis^7–10^. However, as the hardware foundation of flexible electronics, most integrated electronic (IC) systems are based on printed circuit boards (PCB), which have to be attached to the human body in an adorned and patch-like manner, as rigid boxes or bendable bandages^11–15^. The healthcare electronics with the two-dimensional structure of traditional PCB devices, even though bendable or stretchable, still lack features that afford comfortability to the wearer such as air permeability and water absorption akin to clothing that humans naturally wear which are soft and deformable, breathable, durable, and washable^16–18^. Therefore, these types of equipment were psychologically burdensome and hindered long-term use especially for use in healthy lifestyle management.

Progress has been made in the pursuit of a better way of merging electronics with common cloth^19–22^, and even towards an ultimate dream of “fabric computer”, which would be worn as a body-fitted cloth, yet accomplish a complete set of functions, including environmental and physiological perception, information transduction, numeral and logical computing and wireless data-transmission^23,24^. Recently, energy and sensor devices in the shape of fibers or fabric have gained wide attention^25–30^. However, most integrated electronic circuits of these flexible devices were still built on rigid PCB modules. A game-changing solution for the issue is to develop a woven-textile alternative to PCB, which is not only constructed based on all fiber-shaped electric elements but also possesses a complete circuit within its woven pattern borne from the weaving process.

Herein, we present a non-printed integrated-circuit textile (NIT) via weaving method with both wireless monitoring and logical computing capabilities for continuous on-body AI monitoring. All the devices, including transistors, sensors, diodes, solar cells, and batteries were assembled along polymer wires or at their cross-nodes and then integrated as IC function modules for sensing, signals amplifying, logic computing, wireless transmission, and uninterrupted power supply into a cloth-like system by textile weaving. With no external power or signal cable connection, this single piece of fabric is tender and comfortable and can monitor routine healthcare tasks continuously around the clock and encode logical codes for emergency assistance.

## Results

### Structural design of the non-printed integrated-circuit textile

**Fig. 1:**
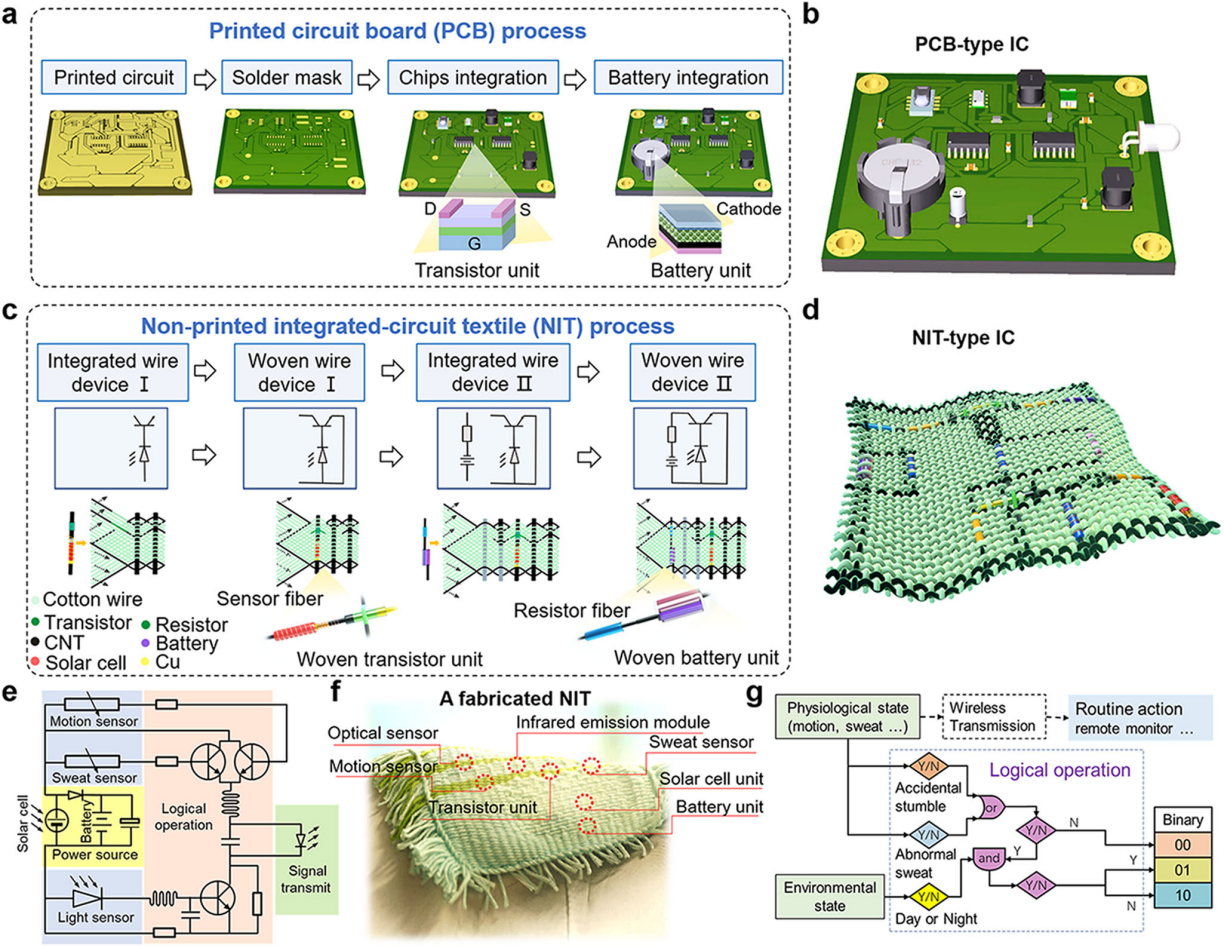
The design of the non-printed integrated-circuit textile. **a, b** The traditional printing circuit board (PCB) process and a PCB-type integrated circuit (IC). **c, d** Schematic illustration of the non-printed integrated-circuit textile (NIT) process and an NIT-type integrated-circuit (IC). **e** The electric circuit design for a typical NIT-type IC. **f** The photograph of a fabricated NIT with a typical NIT-type IC. **g** Function illustration of logical and wireless data-sending operation for a typical NIT.

As shown in Fig. 1a, b, traditional integrated circuits (IC) were fabricated via a printed circuit board (PCB) process, by setting device elements on a flat plastic board or slice. For the first step, the designed circuit with an appearance of the printed pattern was prepared based on a copper-clad laminate by etching methods. For the second step, the solder mask and drilling were carried out to prepare for the subsequent welding of components. For the third step, the chips composed of transistor units were integrated on the board via welding methods. Each unit of a typical transistor device was assembled by stacking conductive and semi-conductive materials layer-by-layer, to form a sandwiched structure. At last, other components, like the battery units, were assembled on the board. A typical battery device was also assembled by stacking conductive and functional materials layer-by-layer, to form a flat structure or cylindrical structure. Such a PCB process has been massively reproduced in the electronic industry, to form complete integrated circuit systems inside computers and cell phones (Fig. 1b).

In contrast, a typical fabrication process for constructing a partial circuit inside a textile non-printed integrated circuit, composed of transistors, sensors, resistors, and batteries, etc., is quite different from the existing PCB process on soft plastics (Fig. 1c), of which the fabrication details are described in Supplementary Note 1 and compared in Supplementary Note 2. A close equivalent to this process is the Jacquard weaving technology employed in the textile industry, which can be denoted as a non-printed integrated-circuit textile (NIT) process. During this weaving process, all the devices were assembled along polymer wires or at their cross-nodes (Supplementary Figs. 1–10) and then integrated as IC function modules into a cloth-like system by textile weaving.

During the weaving process, we pre-fixed various types of warp strings on the fixed shuttle and aligned them one-by-one according to the previous circuit design. And, one end of each warp was fixed on a specially designed harness, so that all the warps were tilted up-and-down alternately to form a clamping opening. For the first step, we fabricated a wire-type integrated device containing both the optical sensor and the gate electrode of the transistor by coating two separate sections of MWCNT next to one conductive end of the light sensor fiber (Supplementary Note 1: Weaving process for the non-printed integrated-circuit textile; Supplementary Fig. 11). We then deposited a PEDOT: PSS layer between the two MWCNT sections, to serve as the gate electrode of the transistor. This wire-type integrated device could be fed into the clamping opening of the warps as a weft. Subsequently, after feeding the wire-type integrated device into the clamping opening, the tilting direction of each warp would be alternately changed to form a new clamping opening. Another PEDOT: PSS layer on the warp would then contact with the gate electrode of the transistor to form an intercross node, which could serve as a complete transistor unit with three terminal ends. For the third step, another wire-type integrated device containing both resistor unit and battery unit was fabricated. After assembling a Zn-MnO_2_ type battery fiber along a cotton wire, we coated two separated sections of MWCNT next to the MWCNT end of the MnO_2_ electrode of the batter (Supplementary Note 1: Weaving process for the non-printed integrated-circuit textile; Supplementary Fig. 12). Then, a layer of activated carbon was coated with the two MWCNTs to form a resistor fiber unit connected to the battery. The wire-type integrated device containing both resistor fiber and batter fiber could be also fed into a clamping opening of the warps as a weft. For the fourth step, after the wire-type integrated device was fed into the clamping opening, the tilting direction of each warp would alternately change and form a new clamping opening. Two MWCNT sections coated on different warps would then contact simultaneously with different conducting terminals of the battery and resistor, which would electrically collect the integrated device into the circuit. By repeating the weaving cycle, other weft containing other components, such as stress sensor and capacitors, etc., could be woven into the NIT-type IC, to enlarge the integrated circuit (Supplementary Figs. 13, 14). Each fiber device could be encapsulated in a PMMA layer or further winded with fabric wires for better comfortability (Supplementary Fig. 15).

As indicated in Fig. 1d. with a completely different appearance from a conventional PCB-based IC system, the soft NIT process possessed a fully interwoven structure, yet functioned as an independent IC system, with texture-type IC function modules of sensing, logic operation, wireless communication, and power supply.

Besides, the fiber weaving process was amenable to integration with different devices in one wearable system. Notably, different types of fiber devices could be fabricated separately at their optimized fabrication conditions, and then woven together into one piece. In addition, the woven texture of the circuit could be fabricated in a scalable manner, by utilizing weaving equipment like that in the textile industry (Supplementary Note 1).

As a prototype for the NIT-type IC, we fabricated a complete NIT following the circuit design in Fig. 1e and presented it as Fig. 1f. The prototype of this body-fitting and common-looking fabricated NIT intelligent textile possessed excellent breathability and moisture permeability.

As indicated in Fig. 1f, the NIT included parts of sensor, logical operation, wireless communication, and power supply. Powered by a textile-type module of solar energy harvesting and storage, it could ceaselessly convert information for body movement, sweat, and ambient light into electric signals by three sets of textile-type sensors. These signals were then amplified by the textile-type conversion modules for real-time wireless sending, or encoded into binary logical codes for different emergency conditions by a logic operation module based on textile-type transistors. Wireless data sending could be achieved by either an infrared emission fiber or even a more complex frequency-modulation or digital communication circuits.

As indicated in Fig. 1g, for the fabric-type logic operation module, smart logical judgments could be made based on the three sets of sensor signals. If there was no movement and abnormal sweating, the fabric would judge the situation as “normal”. At this time, whether day or night, the status is encoded into binaries of (00), and there would be no need for emergency aids. When an accidental fall or abnormal sweating occurred, the status was judged as “abnormal”. The high-level risk or low-level risk could be judged in the context of the environmental status, such as day or night. When it was daytime (simulating when the wearer was not alone and assistance was at hand), the abnormal situation could be encoded into binaries of (01) as “low-level” risk. When it was nighttime (simulating when the wearer was asleep), the abnormal situation could be encoded into binaries of (10) for “high-level” risk.

Such a fabricated NIT without any power or signal cable connection could independently and uninterruptedly accomplish tasks of both wireless monitoring for subsequent intelligent data analysis and logical operation for emergency assistance, which can be applied as a soft type of hardware for on-body AI healthcare, a 24/7 private “nurse” hidden in cloth.

### The textile-type logical operation modules

**Fig. 2:**
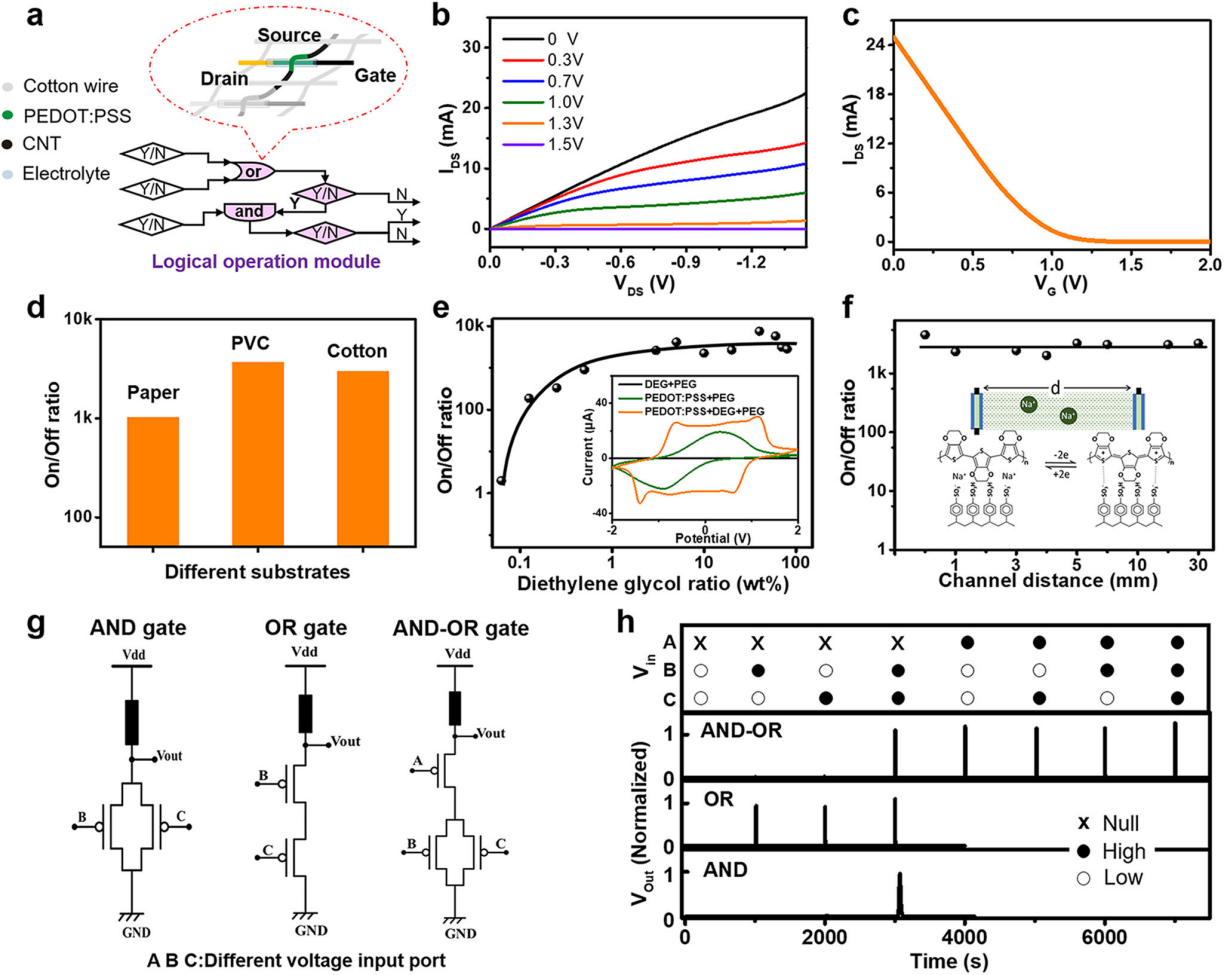
The performance of the logical operation module. **a** The device structure of the woven transistor unit in a logical operation circuit. **b** Electric characteristics of a woven transistor unit. **c** Transfer characteristics of a woven transistor unit. **d** The on/off ratio of the transistor based on different substrates. **e** The on/off ratio of the woven transistor unit with different diethylene glycol ratio in the PEDOT: PSS layer. Insert is the cyclic voltammetry scanning between the gate electrode and the source electrode for PEDOT:PSS layer with different compositions. **f** The on/off ratio of the fabric-type transistor with different channel distance. **g** the logic circuit design for AND gate, OR gate, AND-OR gate. **h** The logic function characteristics of the three types of logic gates.

Transistors are the fundamental building blocks for intelligent operation circuits of signal conversion, amplification, and logic computing. As shown in Fig. 2a, a typical logical operation module with an OR gate was composed of two transistors, and one interlaced transistor node consisted of two PEDOT: PSS coated fibers, which were separated from direct contacting by cotton wires, and were electrically connected at the junction by gel electrolyte (Supplementary Note 1: Fabrication of the fabric-type transistor; Supplementary Figs. 4, 5). The electrical property of the transistor is indicated in Fig. 2b, c. It showed typical characteristics of the depletion-mode transistor. By increasing the gate voltage from 0 V to 1.5 V, a preliminary transistor indicated a conductivity drop of more than 3000 times, which could be attributed to the on state and the off state, respectively. Besides, as shown in Fig. 2d, this type of transistor could be assembled via a simple wet coating method on different substrates, such as paper, PVC, and cotton wires. All of them showed on/off ratios of greater than 3 orders of magnitude, and a pinch-off voltage of about 1.1 V (Supplementary Fig. 16), bringing about convenience for massive fabrication at a low cost.

It is noteworthy that the operation of the transistor relied on the conductive PEDOT: PSS films^29–32^ and ion dopants, which would endow the fiber transistor in the woven logical module with better robustness for bending or stretching. As demonstrated in Fig. 2e and Supplementary Fig. 17, the doping/de-doping process of Na-ions could be further enhanced by adding diethylene glycol (DEG) in the PEDOT: PSS layer as a secondary dopant. With a secondary dopant content of >1%, the on/off ratio was significantly promoted by more than 3 orders of magnitude. We further investigated the working principle by electrochemical analysis of the gate electrode and the source electrode (Fig. 2e and Supplementary Fig. 18). By adding DEG into PEDOT: PSS layer, two additional pairs of redox peaks appeared symmetrically around 1 V and −1 V, in addition to the reversible electrochemical adsorption and desorption peaks of Na ions in the shallow layer of PEDOT: PSS^33,34^. These additional redox peaks may be attributed to the conversion of PEDOT nanocrystals from a face-to-face structure to a layered structure (Supplementary Fig. 19) with lower charge-transfer barriers, which allowed more in-depth doping/de-doping of Na-ions^35,36^.

As shown in Fig. 2f, the textile-type transistor exhibited better robustness for on/off performance during bending or stretching, owing to the much longer carriers-transfer distance of Na ions than that in conventional semiconductor transistor. For a large electrode displacement of 3 cm, the ionic impedance change of the gel electrolyte was less than 2 KΩ (Supplementary Fig. 20), which only took a small portion of the total impedance (>20 KΩ) between gate and source electrodes. The insensitive transistor operation to the deformation environmental changes would bring convenience to both large-scale fabrication and practical wearable applications (Supplementary Fig. 21).

As indicated in Fig. 2g, h, more complex logic operation circuits, including AND gates, OR gates, and AND-OR gates were fabricated based on the textile-type transistors. For all of them, low vs high output logic levels (logic “0” vs logic “1”) could be achieved with correct logic functions, which exhibited a potential towards smart integrated circuits for making intelligent logic judgments.

### The textile-type sensor modules

**Fig. 3:**
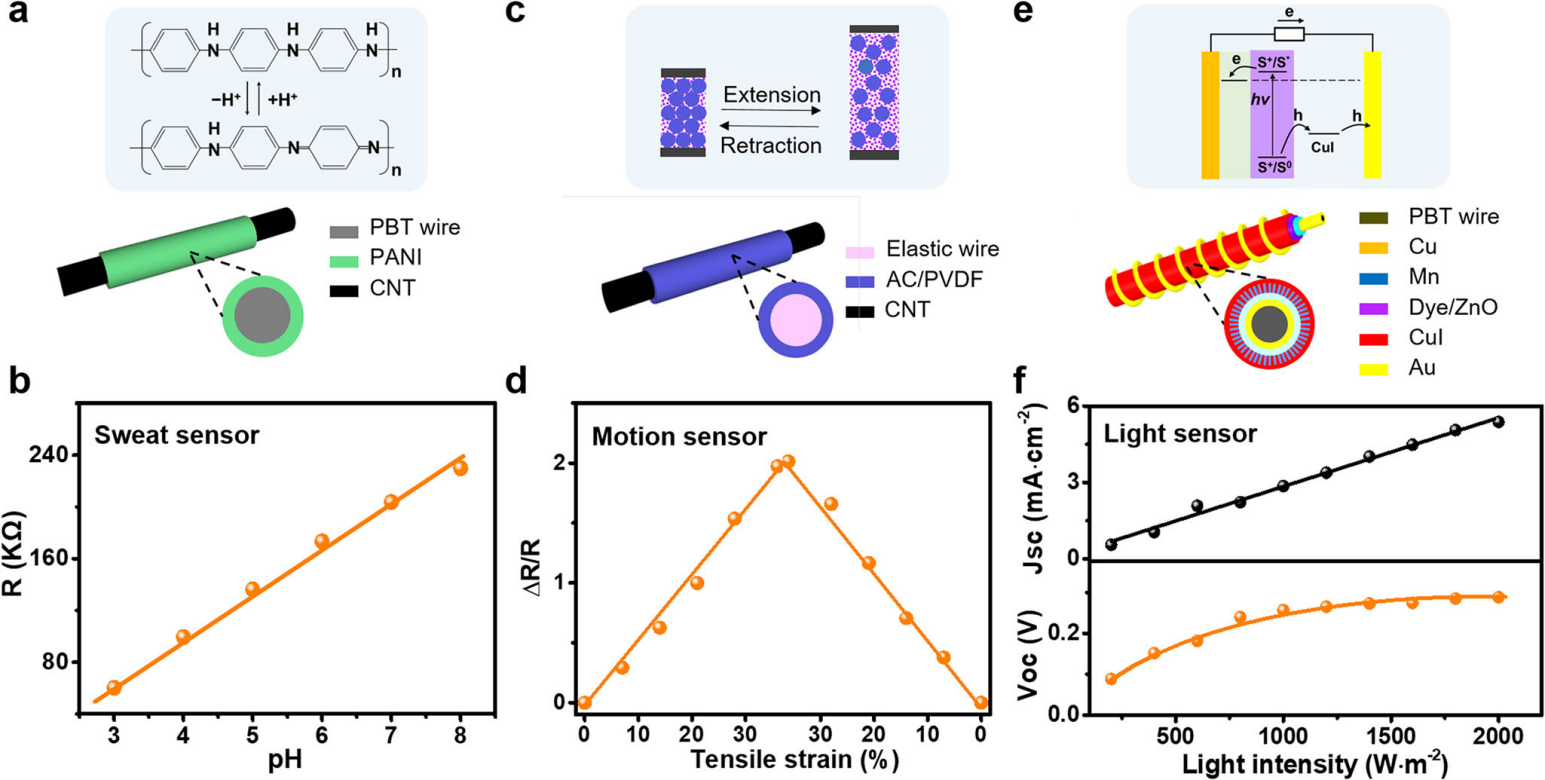
The performance of the sensor module. **a, b** The device structure of the sweat sensor and its sensitivity at different pH values. **c, d** The device structure of the motion sensor and its sensitivity at different tensile strains. **e, f** The device structure of the light sensor and its sensitivity at different light intensities.

For the sensor modules, we assembled a fiber-type sweat sensor and weaved this sensor with strain and light-sensing fibers for simultaneous body-health and environment monitoring (Fig. 3 and Supplementary Figs. 22–24). Among them, the sweat sensor was crucial for probing biofluid from deeper in the body which may carry diverse molecules ranging from small metabolites to larger proteins. Integrating this with the profile analysis of body movements and environmental conditions would provide useful information for effective medical diagnosis.

Figure 3a shows the structure and working principle of the fiber-type sweat sensor. Polyaniline (PANI) is a conductive polymer^37^ that, together with CNT^38,39^, can be used for making robust and sensitive sensors. The fiber-type sweat sensor was assembled by the in-situ growth of PANI films on the PBT wire between two coaxially coated sections of MWCNTs. The pH sensor worked based on the reversible protonation/deprotonation process inside the PANI layer, which could lead to a reversible conversion of the PANI molecule between emeraldine salt and emeraldine base. Figure 3b and Supplementary Fig. 25 illustrate the resistance responses of an optimized wire-shaped pH sensor, measured within the pH of 3 to 8, in accordance with the pH value range for the sweat of both sub-healthy and healthy humans. A reversible linear relationship between resistance and pH value with a sensitivity of 40 kΩ was achieved.

Figure 3c shows the structure and working principle of the fiber-type motion sensor. We assembled this sensor by coating an activated carbon/polyvinylidene fluoride (AC/PVDF) layer onto the elastic wire, which was electrically led out by conductive MWCNTs. When the sensor was stretched under an external force, the average distance between any two AC particles inside AC/PVDF layer increased resulting in a change in resistance. Figure 3d and Supplementary Fig. 26 show the representative resistance responses of an optimized wire-shaped piezoresistive sensor, measured in 0–40% of tensile strain, in accordance with the range of tensile strain for the stretching of sportswear during running. A reversible linear relationship between the resistance and the tensile strain was observed, which showed a ΔR/R of 2 at a 40% tensile strain.

Figure 3e shows the structure and working principle of the fiber-type light sensor. We fabricated this sensor by winding an Au wire around a fiber-shaped electrode composed of PBT/Cu/Mn/ZnO/Dye/CuI. When excited by photon, a pair of hole and electron would form on the ZnO/Dye/CuI interface, and then directionally separated to form a current or bias as a sensor output. Its current and voltage output would change along with the light intensity. Figure 3f and Supplementary Fig. 27 show both the open-circuit voltages (Voc) and the short-circuit currents (Isc) of light sensors within a range from dark to 2000 W/m^2^, which would cover possible illumination environments for normal human lives. A reversible linear relationship between short-circuit current and light intensity with a sensitivity of 0.03 A/W was observed, while the voltage tended to a stable value for light intensity above 800 W/m^2^.

Furthermore, different types of signals from different types of sensors could be regulated to match with standard electrical output, by designing different amplifier circuits. As indicated in Fig. 1e and Supplementary Fig. 28, for both the strain sensor and the pH sensor, the resistance variation could be regulated into a voltage signal with higher impedance matching capability, by designing a textile-type common-collector amplifier circuit based on transistors. Also, the power output of the light sensor could directly turn off the carrier channel of the transistor.

### The textile-type power modules

**Fig. 4:**
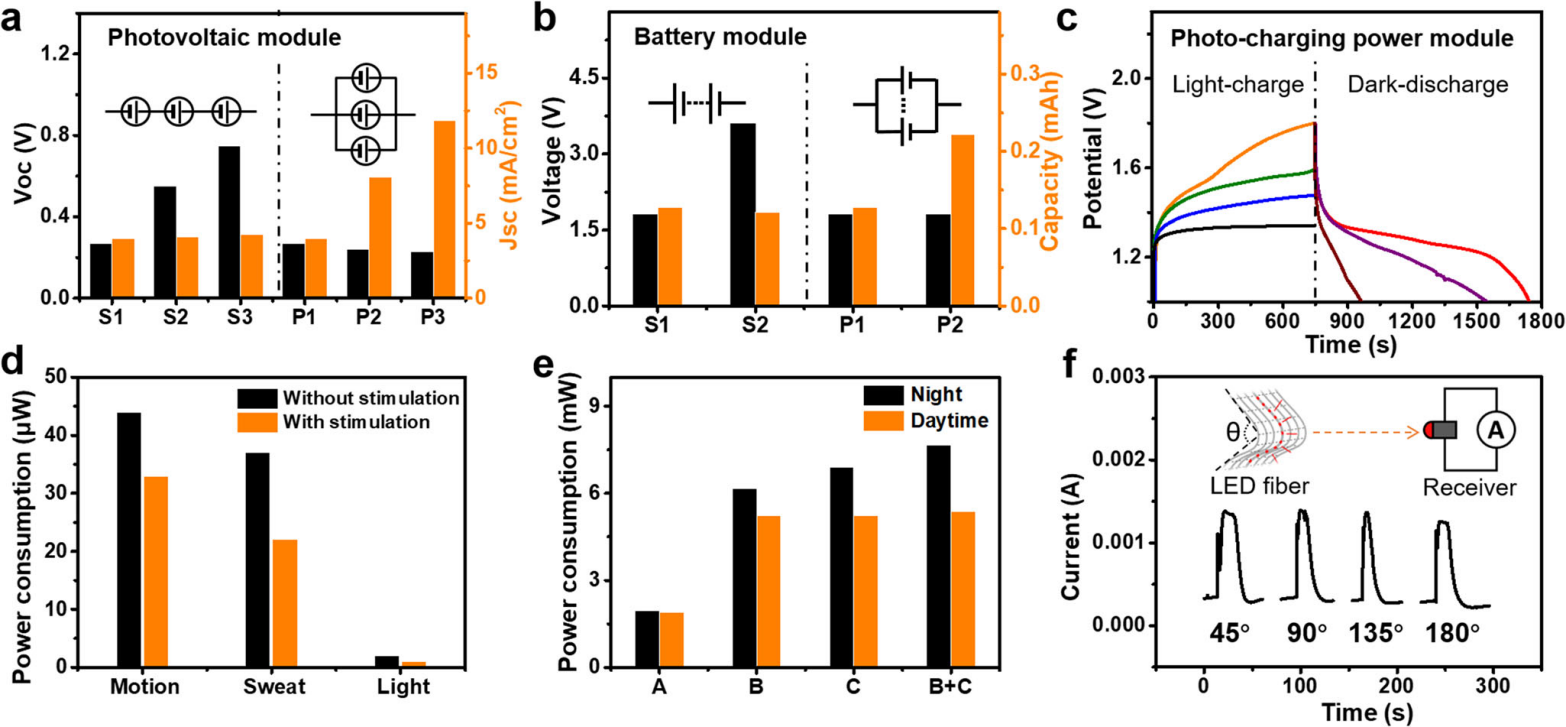
Performance of the textile-type power modules. The performance of textile-type photovoltaic (**a**) /battery (**b**) module connected in series vs in parallel. **c** The photo-charge and dark-discharge performance by integrated textile-type photo-charging power modules. **d** The power consumption of different sensors without or with stimulation. **e** The power consumption at different stimulations in night-time or day-time: A. no stimulation, B. body moving, C. sweating. (**f**) The fabric data-sending performance at various bending angles.

The flexible photo-rechargeable fabric woven in the NIT presents a self-powered energy solution for frequent charging, instead of traditional batteries with a rigid structure. To drive logic operation and sensor modules continuously, both a textile-type photovoltaic energy-harvesting component and rechargeable Zn/MnO_2_ battery fiber were woven into the intelligent textile to form a sustainable and photo-rechargeable power supply (Supplementary Fig. 29 and Supplementary Fig. 30). In Fig. 4a and Supplementary Fig. 31, we tested the output of photovoltaic units connected in series and in parallel. In series connections, the Voc increased linearly with fiber photoanode numbers and the Isc remained constant. For the parallel connection, its Isc increased linearly with the number of fiber photoanodes, while the Voc remained constant. As shown in Fig. 4b and Supplementary Fig. 32, each optimized Zn/MnO_2_ battery fiber could reach up to a capacity of 190 mAh/g. Larger voltage and capacity could be achieved by connections in series and parallels, respectively. In addition, to prevent the self-discharge between the photovoltaic module and the battery module, the unidirectional blocking diodes were applied (Supplementary Fig. 33). For reducing the noise from the power supply to signal conversion, we introduced wire-shaped polymer dielectric capacitors with a capacitance of 100 pF (Supplementary Fig. 34) to form a typical capacitance filter circuit. As shown in Fig. 4c, a complete photo-rechargeable module can achieve 1.8 V beneath the sunlight, which could realize an 18 min continuous 0.1 mA electric power output.

The hybrid energy module could provide enough power for other modules in the textile sustainably and stably. The power consumptions of each sensor module under stimulation or not are summarized in Fig. 4d. To give notable sensor responses on the body movements and sweating, both the motion and sweat monitoring modules required no more than 45 μW of power consumption. Moreover, the active light sensor could directly drive the transistor by harvesting solar energy with no power consumption. Furthermore, as indicated in Fig. 4e, the infrared emission signal of the wire-type LED reached up to 6–8 mW for seconds at stimulation and then dropped or stayed at the stand-by mode. Besides, wireless optical communication only reached its peak power consumption in emergency conditions. A textile containing a photovoltaic component of 3 cm × 5 cm and a battery fabric component of 2.5 cm × 5.2 cm, would continuously complete a set of tasks without needing to connect to an additional charger.

Finally, the textile could accomplish its task by wireless sending data or logical binaries based on the infrared LED wire (Supplementary Table 1), as a completely independent system without connecting to any additional wireless communication PCB module. The communication operated normally even at a bending angle of 180^o^, showing good flexibility (Fig. 4f). It may be attributed to the low angle dependency of the wire-type infrared LED (Supplementary Fig. 35). As shown in Supplementary Fig. 36, after up 5000 cycles of bending, the wireless communication modules still functioned properly, which is very promising for on-body applications. Meanwhile, the textile has good waterproof and thermostable performance, which is not affected by environmental humidity and temperature (Supplementary Fig. 37). With a photo-rechargeable energy textile based on a detailed power consumption analysis, the woven circuit textile would completely provide energy for any other modules among the NIT.

### Function demonstration of the non-printed integrated-circuit textile

Ideally, the NIT has demonstrated capabilities of both wireless physiological monitoring and early warning for simulated emergencies of diabetes, hypoglycemia, metabolic alkalosis, etc., which could potentially serve as a future on-body AI hardware.

**Fig. 5:**
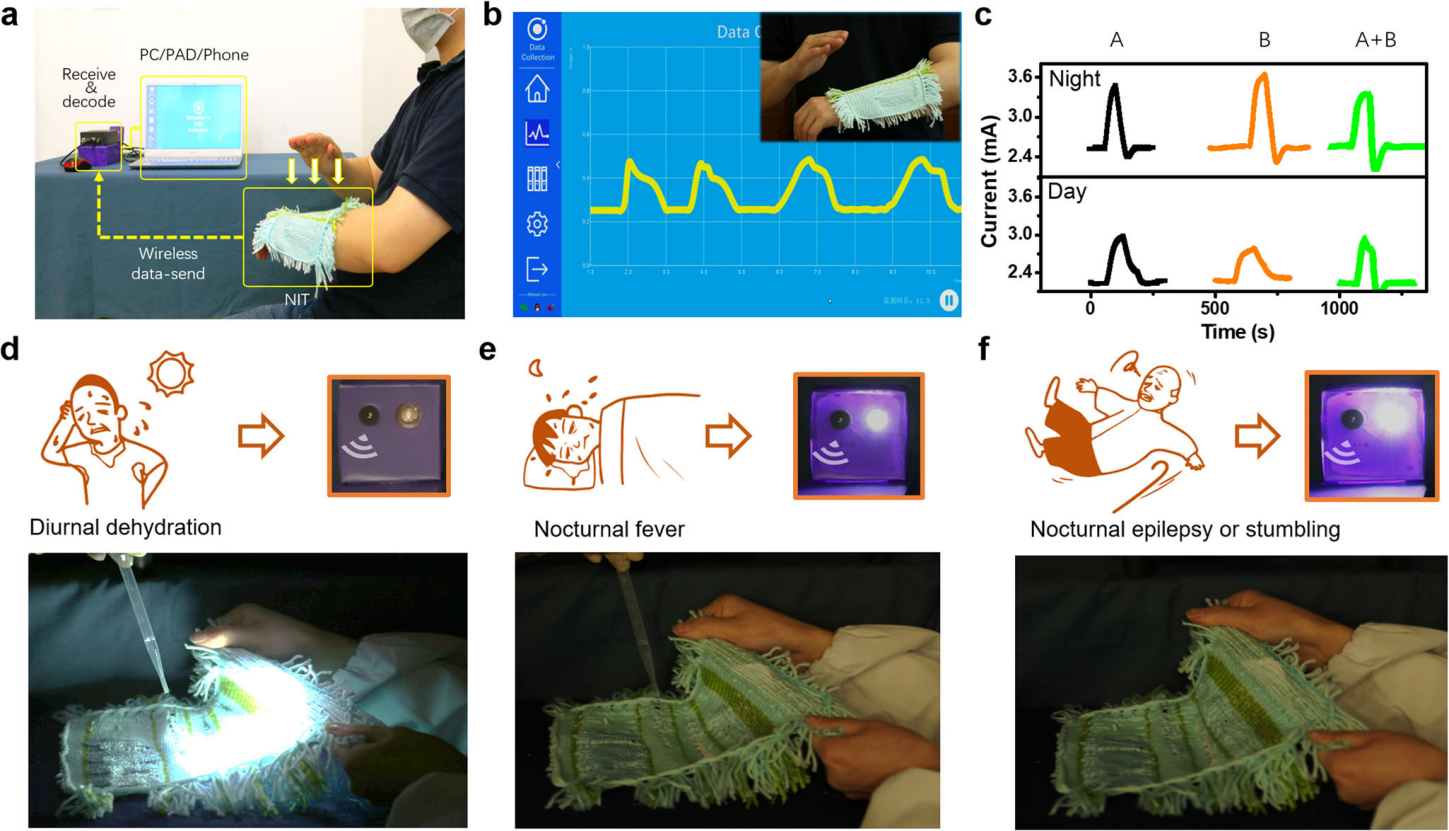
Function verification of a prototype for both wireless data-sending and emergency alarms. **a** An illustration of wirelessly sending body-motion data from the NIT to a tablet PC in the lab. **b** The wireless data profile from the fabric. **c** The received signal level for different scenarios in night-time or day-time: A. body moving, B. sweating. The fabric can send different logical binaries by itself to determine alarm types for different simulated emergencies: (**d**) sound only alarm for abnormal pH value change under light illumination, (**e**) both sound and light alarm for abnormal pH value change in the dark, (**f**) both sound and light alarm for the stretching or hitting on the body in the dark.

For the representative application of wireless physiological monitoring, the intelligent textile could receive all three signals of sweat, motion, and light, and send them out wirelessly via optical signals. As indicated in Fig. 5a, we demonstrated a typical setup in the lab. A wrister-like intelligent textile without any wire connection was put on the arm. When it was patted or stretched randomly, it would detect the action, and send corresponding signals wirelessly to a remote receiving module connected with a tablet PC (Supplementary Video 1). Consequently, the synchronous signals were recorded by the computer (Fig. 5b).

The other representative application is as an intelligent “nurse” hidden in common-looking clothes for emergency surveillance and response. It would detect the dangerous situation of diurnal dehydration, nocturnal fever, and nocturnal epilepsy or stumbling, and send out corresponding logic code to the remote terminal to warning doctors or family members for help. As summarized in Fig. 5c, the NIT would detect typical basic actions of the body movement, sweating and their combined actions, and then, make logical judgments by emitting infrared LED signals with different intensities for either daytime or nighttime.

For simulated medical emergencies combining more basic actions, the emission intensity of the NIT could be encoded into three levels of 0, 1, 2, or more. For the normal cases, it kept to an emission intensity of level 0. In case of lower-risk emergencies during the daytime, such as daily dehydration and excessive exercise, the emission intensity would reach level 1, to remind the nurse for in-time advice. In case of emergencies with higher-risk levels, like nocturnal fever and nocturnal epilepsy during the nighttime, the emission intensity would reach level 2, to trigger a different type of alert for emergency aids.

As simulated in Fig. 5d–f, a patient with a history of chronic diabetes needs special care for his or her sweating and body motion status. Complications, like hypoglycemia and epilepsy usually companied with an increased pH value of sweat and abnormal body moving, which can be life-threatening but in need of 24/7 monitoring for achieving early alarm^40–42^. COVID-19 patients also require extensive healthcare^43,44^. Together with traditional and ongoing theranostics development, we will be able to provide better human health services^45–55^. Typical situations were simulated to demonstrate the capability of early warning in an emergency.

First, when abnormal pH value of sweat was detected by the fabric during the daytime, it may suggest situations of daily dehydration, excessive exercise, and so on. Nurses who get this type of alarm would promptly give patients advice, like avoiding strenuous exercise or an extra supply of sugar. As indicated in Fig. 5d, our fabric can only trigger the sound alarm, by giving signals of level 1.

Second, when abnormal pH value of sweat was detected by the fabric during the nighttime, it may suggest situations of hypoglycemia, nocturnal fever, metabolic alkalosis, and so on. Nurses or family members who get this type of alarm would immediately go to check the status of the patient, or even call the emergency center for medical aids. As indicated in Fig. 5e, our fabric can trigger both the sound alarm and the light alarm simultaneously, by giving different signals of level 2.

Third, when abnormal body moving was detected by the fabric during bedtime, it may suggest situations of nocturnal epilepsy or the patient experiencing a fall. Nurses or family members who get this type of alarm would also immediately check the status of the patient, or give medical aids under the remote instructions of a doctor. As indicated in Fig. 5f, our fabric can also trigger both the sound alarm and the light alarm, by giving signals of level 2.

More demonstrations for emergency alarms were also provided in Supplementary Video 2. For all of them, the remote alarms can be triggered properly and wirelessly, by the same piece of common-looking fabric.

## Discussion

In summary, we have presented a non-printed integrated-circuit textile with both wireless monitoring and logical computing capabilities. All the device components were assembled and integrated along polymer wires or at their cross-nodes during weaving. A prototype has been fabricated as an independent textile without any external power or data cable connection. It has demonstrated its capability of multiplexed sensing, signal amplifying, logic computing, and wireless data transmission. Such a soft AI hardware can be worn on a human body as a common cloth, yet function as a 24/7 private AI nurse, for routine healthcare monitoring around the clock or emergency assistance, a might even one day lead to the success of fabric-like computer.

## Methods

### Fabrication of wire-type tensile stress sensors

The AC slurry was made from a 3:1 weight ratio of AC (Kuraray, YP-50f): PVDF mixture inside the 1-methyl-2-pyrrolidone solution. It was further deposited on an elastic wire, of which the two ends were respectively stuck to two cotton wires. Each end of the AC-coated elastic wire was led out by coaxially coating a layer of MWCNTs, to form two conductive terminals on the cotton wire (Supplementary Note 1: Fabrication of the wire-type tensile stress sensor; Supplementary Fig. 1).

### Fabrication of wire-type pH sensors

The polymer wire was put into the aniline (0.056 mol/L) / hydrochloric acid (0.35 mol/L) solution for 30 min under ultrasonic. It was then reacted with (NH_4_)_2_S_2_O_8_/hydrochloric acid (0.35 mol/L) under an ice bath for 3 h, to coat a layer of polyaniline (PANI). Each end of the PANI layer was led out by coaxially coating a layer of conductive MWCNTs to form two conductive terminals (Supplementary Note 1: Fabrication of the wire-type pH sensor; Supplementary Fig. 2).

### Fabrication of wire-type optical sensors

A copper layer was deposited onto a polymer wire (diameter = 0.26 mm) via a chemical plating process in the mixture of sodium hydroxide (0.2 M), formaldehyde (0.1 M), EDTA (0.05 M C_10_H_14_N_2_Na_2_O_8_·2H_2_O), and copper(II) sulfate (0.03 M) (See more details in Supplementary Note 1: Fabrication of the wire-type optical sensor). Then, the Mn layer was deposited on the polymer/Cu wire via the electroplating process from an aqueous MnSO_4_ solution (0.059 M) (See more details in Supplementary Note 1: Fabrication of the wire-type optical sensor). After electroplating, the polymer/Cu/Mn wire was washed and then dried. Then, ZnO-nanowires were deposited by reacting the mixture containing Zn(CH_3_CO_2_)_2_ (0.01 M) and hexamethylenetetramine (0.01 M) at 95 °C overnight. After being taken out and cleaned with deionized water, the ZnO layer was dried in a vacuum and soaked in the N719/C_2_H_5_OH solution for one day. Then, the CuI layer was coated by brushing the CuI/CH_3_CN solution (nitrogen environment, 130 °C). The wire-type optical sensor was prepared by twisting Au/Cu wire around the CuI coated wire electrode and sealed in polymethyl methacrylate (PMMA). (Supplementary Note 1: Fabrication of the wire-type optical sensor; Supplementary Fig. 3).

### Fabrication of fabric-type transistors

A section of conductive PEDOT: PSS-1000 (Xi’an Polymer Light Technology Corp.) containing 10 wt% of diethylene glycol and 0.5 wt% of PEG-2000 was coated on the cotton wires for an optimized doping condition. Each end of the PEDOT: PSS section can be led out by coaxially coating a layer of MWCNTs as the conductive terminals. Each device node of the fabric-type transistor was constructed by two intercrossed PEDOT: PSS/cotton wires. A cotton wire was tangled on one of the PEDOT:PSS/cotton wire as the spacer. The transistor node was then contacted by dropping gel electrolyte containing 8 wt% sorbitol, 33 wt% PSS, 0.1 M sodium perchlorate, 12 wt% glycol, and deionized H_2_O using micro-syringes, and was encapsulated by coating a layer of PMMA. More details on a typical weaving process for a fabric-type transistor circuit containing more than one transistor nodes were provided in Supplementary Note 1: Fabrication of the fabric-type transistor and Supplementary Figs. 4, 5.

### Fabrication of wire-type polymer dielectric capacitors

PVDF/N-methyl pyrrolidone solution (2.5 g/L) was brush coated on a Cu-coated section of a cotton wire. After dried in the air, a thin copper wire was then winded around the PVDF section, and then connected to one conductive terminal of another device on the same cotton wire (Supplementary Note 1: Fabrication of the wire-type polymer dielectric capacitors; Supplementary Fig. 6).

### Fabrication of wire-type resistors

Resistance is fabricated by coating a layer of AC (Kuraray, YP-50f) onto cotton wires, repeatedly, via the self-designed brush coating machine. Both ends of the AC section were led out by coaxially coating a layer of MWCNTs, to form two conductive terminals on the cotton wire. The resistance is controlled by the length of the AC section (Supplementary Note 1: Fabrication of the wire-type resistor; Supplementary Fig. 7).

### Fabrication of fabric-type photovoltaic cells

For the photoanode of the fabric-type photovoltaic cell, it was assembled via a similar process as that of the photoanode for the optical sensor, while the reaction solution for ZnO-nanowire growth employs the Zn(CH_3_CO_2_)_2_ (0.03 M) and hexamethylenetetramine (0.03 M) solution, which will lead to a longer length of ZnO nanowire. After coating the CuI layer, Au-coated Cu wires were selected as the counter electrode for fabric-type photovoltaic cells, which were then woven with wire-type photoanodes via a shuttle flying process. More details on a typical weaving process for a fabric-type photovoltaic cell were provided in Supplementary Note 1: Fabrication of a fabric-type photovoltaic cell and Supplementary Fig. 8.

### Construction of Zn/MnO_2_ wires

The 2:7:1 weight ratio carbon black (CB), MnO2, and polyvinylidene fluoride (PVDF) mixture in 1-Methyl-2-pyrrolidinone was applied for the production of the MnO2 slurry by a hydrothermal method^25^. Wire-type MnO_2_ electrode was then prepared by coating the MnO_2_ slurry onto PET/MWCNTs wire (Supplementary Note 1: Construction of Zn/MnO2 Wires; Supplementary Fig. 9). It was then aligned with a PET/MWCNTs/Zn wire to form an electrode pair. The electrode pair was buried in a layer of aqueous gel electrolyte containing ZnSO_4_ (2 M), MnSO_4_ (0.4 M), and polyvinyl alcohol (100 g/L). After solidification, the battery wire was encapsulated in a layer of PMMA.

### Fabrication of fabric-type infrared light-emission diodes

Two thin Au wires (diameter = 0. 033 mm) with a distance of <1 mm were stuck on a polymer strip of 1 mm in width. Then, infrared chips (A1GaAs, SLLT6393A, Shenzhen Shenlan Technology Co., Ltd) with two electrode terminals (1.6 mm × 0.8 mm × 0.6 mm) were aligned in a head-to-tail direction and stuck one-by-one on the back of the polymer strip. Their two electrode terminals were electrically connected to the two Au wires by conductive paste, respectively, to achieve the connection in parallel. After sealed by transparent polymer, it can form an infrared light-emission cable (Supplementary Note 1: Fabrication of the wire-type infrared light-emission diodes; Supplementary Fig. 10).

### Weaving process for the non-printed integrated-circuit textile

The weaving process includes two steps. The first step is the fabrication of integrated function strings composed of different fiber-shaped electrode units or device units. The second step is the circuit weaving of NIT by employing integrated function strings as warp or weft.

For the first step of fabricating integrated strings, different types of integrated function strings were fabricated, and each integrated function string was composed of two or more as-mentioned electrode units or device units on one fiber substrate in a section-by-section way. These integrated function strings were later directly used as warps or wefts during weaving. For example, a typical integrated function string containing a PBT/Mn/ZnO/dye/CuI/Au optical sensor and a PEDOT: PSS gate electrode of the transistor was fabricated on one cotton wire (Fig. 1c and Supplementary Fig. 11). During which, the PEDOT: PSS gate electrode was assembled next to the optical sensor on the same cotton wire and was connected to the Au electrode of the optical sensor. The PEDOT: PSS gate electrode section can later serve for weaving a complete transistor device node in the circuit woven step. Besides, with a similar approach, another type of integrated function string containing both resistor unit and battery unit as presented in Fig. 1c, can also be fabricated (Supplementary Note 1: Weaving process for the non-printed integrated-circuit textile; Supplementary Fig. 12).

For the second step of circuit woven, not only interwoven-type device units can be assembled on the nodes between warp and weft, but also the circuit connection and integration can be accomplished through the woven texture. On one hand, typical interwoven-type device units, like the transistor node presented in Fig. 1c, can be assembled by weaving. During which, integrated function string containing an optical sensor and gate electrode as the weft, was woven with another PEDOT: PSS wire electrode as the warp. When the weft and the warp were contacted together, a complete transistor node was formed, which was then sealed in an aqueous gel electrolyte of 8 wt% sorbitol, 33 wt% PSS, 0.1 M sodium perchlorate, 12 wt% glycol, and deionized H_2_O.

On the other hand, a typical circuit connection process, such as the circuit connection between one integrated resistor-battery string (RB string) and one integrated sensor-transistor string (ST string), can also be accomplished by weaving. During which, the RB string as the weft was interwoven with an MWCNTs-coated wire as the warp. As indicated in Fig. 1c and Supplementary Fig. 13, the resistor on the RB string was connected via the MWCNTs-coated wire to the drain electrode of the transistor on the ST string, while the battery on the RB string was connected via another MWCNTs-coated wire to the sensor on the ST string, which then forms a textile-type amplifier circuit module for regulating sensor signals.

By repeating the cycle, a more complicated NIT includes parts of sensor, logical operation, wireless communication, and power supply, which can be woven out as shown in Supplementary Note 1: Weaving process for the non-printed integrated-circuit textile and Supplementary Figs. 11–15.

### Characterization of structure and performance

Electrochemical performance and electrochemical impedance spectroscopy were obtained using the CHI600E (Shanghai Chenhua) electrochemical workstation. The scanning electron microscope was applied for morphology studies using the JOEL-JSM-7800F. All the resistance calculation in this paper is the resistance value calculated by testing the I-V curve and then by the reciprocal of the slope of the curve. The photovoltaic test was conducted under an AM 1.5 San-Ei Electric XES40 simulator at a calibrated 1000 W/m^2^.

## Supplementary information

Supplementary Information

Supplementary Movie 1

Supplementary Movie 2

Description of Additional Supplementary Files

## Data Availability

All relevant data supporting the findings of this study are available within the paper and Supplementary Information files or from the corresponding author upon reasonable request. A reporting summary for this article is available as a Supplementary Information file, including details on the fabrication of the wire-type tensile stress sensor, pH sensor, optical sensor, transistor, dielectric capacitors, resistor, photovoltaic cell, Zn/MnO_2_ battery wires, and infrared light-emission diodes; Weaving process of NIT textile; Performance of wire-type transistor, pH sensor, tensile stress sensor, optical sensor, photovoltaic textile, Zn-MnO_2_ battery wires, dielectric capacitors, infrared light emission, and NIT textile.
