# Supplementary material for: A non-printed integrated-circuit textile for wireless theranostics
Source: Nat Commun. 2021 Aug 12;12:4876. doi: 10.1038/s41467-021-25075-8 (PMC8361012; doi:10.1038/s41467-021-25075-8)
Supplement: Supplementary file 1 — Supplementary Information [file 41467_2021_25075_MOESM1_ESM.pdf]

Supplementary Information

A non-printed integrated-circuit textile for wireless theranostics

Yuxin Yang[1,2,3#], Xiaofei Wei[1#], Nannan Zhang[1]*, Juanjuan Zheng[4], Xing Chen[4], Qian Wen[1,3], Xinxin Luo[3], Chong-Yew Lee[5], Xiaohong Liu[2], Xingcai Zhang[4,6]*, Jun Chen[7], Changyuan Tao[1], Wei Zhang[2]*, Xing Fan[1,3]*

[1] College of Chemistry and Chemical Engineering, Chongqing University, Chongqing 400044, China

[2] Chongqing Institute of Green and Intelligent Technology, Chinese Academy of Sciences, Chongqing 400714, China

[3] Industrial Technology Research Institute of Chongqing University, Chongqing 400044, China

[4] John A. Paulson School of Engineering and Applied Sciences, Harvard University, Cambridge, MA 02138, United States

[5] School of Pharmaceutical Sciences, University Sains Malaysia, Penang 11800, Malaysia

[6] School of Engineering, Massachusetts Institute of Technology, Cambridge, MA 02139, United States

[7] Department of Bioengineering, University of California, Los Angeles, Los Angeles, CA 90095, USA

[#]These authors contributed equally to this work.

*E-mail: zhangnn@cqu.edu.cn; xingcai@mit.edu; zhangwei@cigit.ac.cn; foxcqdx@cqu.edu.cn

Supplementary Note 1: Details on fiber-device fabrication and weaving process of non-printed integrated-circuit textile.

Materials. PEDOT: PSS-1000 (Xi'an Polymer Light Technology Corp.), activated carbon (AC, Kuraray, YP-50f), sodium polystyrene sulfonate (Aladdin Reagent Co., Ltd). Without special notification, other reagents were purchased from Chengdu Kelong Chemical Reagent Factory. All reagents were commercially available and used as supplied without further purification.

Fabrication of the wire-type tensile stress sensor. An activated carbon (AC) slurry was made from a 3:1 weight ratio of AC (Kuraray, YP-50f): PVDF mixture inside the 1-methyl-2-pyrrolidone solution. It was further deposited on an elastic wire, of which the two ends were respectively stuck to two cotton wires. Each end of the AC-coated elastic wire was led out by coaxially coating a layer of conductive MWCNTs, to form two conductive terminals on the cotton wire. It was then calibrated by recording the resistance changes under different stress.

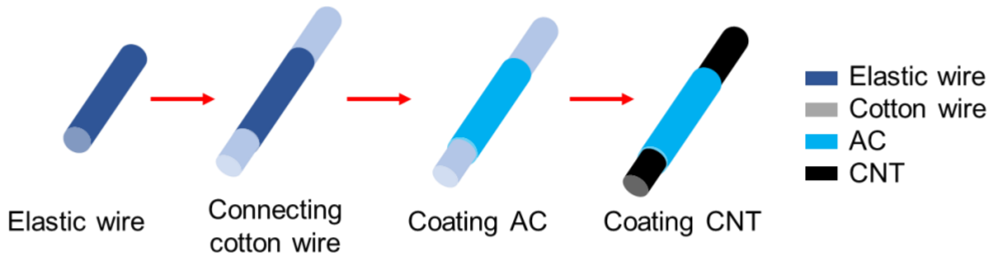

Supplementary Fig. 1 Fabrication process of the wire-type tensile stress sensor.

Fabrication of the wire-type pH sensor. The polymer wire was washed with ethanol and deionized water before use. It was then put into the aniline (0.056 mol/L) / hydrochloric acid (0.35 mol/L) solution, and stayed under ultrasonic for 30min. $(NH_4)_2S_2O_8$ (the mole ratio of $(NH_4)_2S_2O_8$: aniline is 1:1) was dissolved in 20ml of hydrochloric acid (0.35 mol/L) and was then added into the above aniline solution drop by drop under vigorously stirring in an ice bath. The PBT wire coated with polyaniline (PANI) was taken out after reaction in an ice bath for 3 h and dried in air. Both ends of the PANI section were led out by coaxially coating a layer of MWCNTs, to form two conductive terminals on the cotton wire. It was then calibrated by recording the resistance changes in different solutions, of which the pH value was pre-calibrated

by a commercial pH meter (PHS-3C, Shanghai Leici Instrument Co., Ltd).

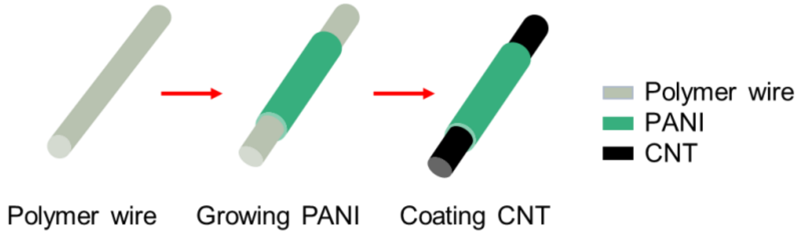

Supplementary Fig. 2 Fabrication process of wire-type pH sensor.

Fabrication of the wire-type optical sensor. Copper was firstly deposited onto a PBT polymer wire (0.26 mm in diameter). During the chemical plating process, the polymer wire was bathed at 50 °C for 40 min in a solution containing $CuSO_4$ (0.03 M), ethylenediaminetetraacetic acid disodium salt (0.05 M), HCHO (0.1 M), and NaOH (0.2 M). Then, a layer of Mn was electroplated onto the PBT/Cu wire. During the electroplating process, the PBT/Cu wire was used as the cathode, whereas $PbSb_{0.0003}Sn_{0.0003}Ag_{0.0003}$ alloy rod (diameter = 5.0 mm) was used as the anode. They were kept at a distance of 1 cm and placed in an aqueous electrolyte containing $MnSO_4$ (0.059 M) and H_2SeO_3 (0.27 mM). After electroplating, the polymer/Cu/Mn wire was cleaned by de-ionized water and dried. Then, ZnO-nanowires were deposited on the polymer/Cu/Mn wire by reacting the mixture containing $Zn(CH_3CO_2)_2$ (0.01 M) and hexamethylenetetramine (0.01 M) at 95°C overnight. After being taken out and cleaned with deionized water, the ZnO layer was dried in a vacuum and soaked in the N719/C_2H_5OH solution for one day. Then, the CuI layer was coated by brushing the CuI/CH_3CN solution (nitrogen environment, 130 °C), which serves as a skin-harmless hole-transmission material. The wire-type optical sensor was prepared by twisting Au/Cu wire around the CuI coated wire electrode and sealed in polymethyl methacrylate (PMMA). PMMA was dissolved in carbon tetrachloride, and used to coat the sensor surface by dipping and pulling. Then the solution can be dried quickly to form a PMMA film.

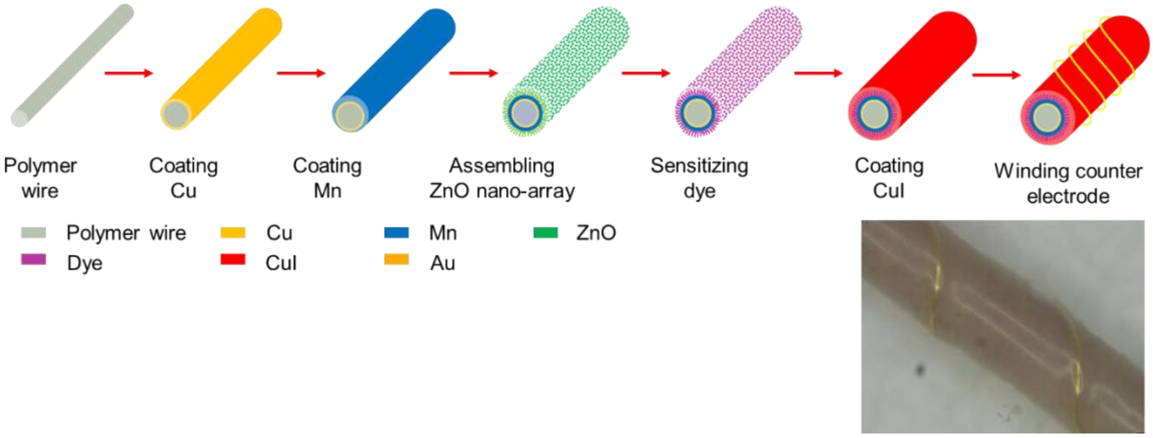

Supplementary Fig. 3 Fabrication process of the wire-type optical sensor.

Fabrication of the fabric-type transistor. A section of conductive PEDOT: PSS-1000 containing 10 wt% of diethylene glycol and 0.5wt% of PEG-2000 was deposited on the cotton wires for an optimized doping condition. Each end of the PEDOT: PSS layer can be led out by coaxially coating a layer of MWCNTs, to form one or two conductive terminals on the cotton wire, as indicated below. The coating process of both the PEDOT: PSS layer and MWCNTs layer can be conducted precisely and segmentally along a long cotton wire, on a self-designed coating machine. Using a similar manufacturing process, we can build a transformer based on PVC or paper.

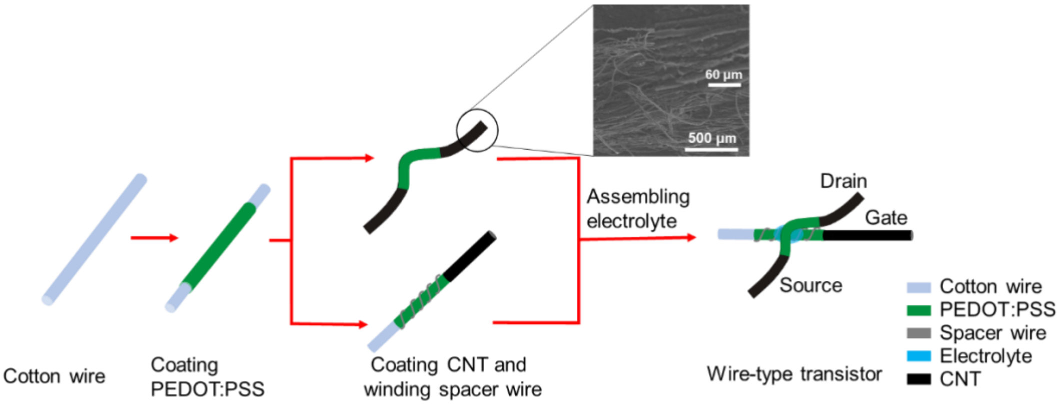

Supplementary Fig. 4 Fabrication process of the wire-type transistor.

For each device node of the fabric-type transistor, one PEDOT: PSS/cotton wire electrode was employed as the warp, and another PEDOT: PSS/cotton wire electrode tangled with a cotton wire as the spacer was employed as the weft, on a weaving machine. By interweaving them

together, the two PEDOT: PSS/cotton wire electrodes can form an intercrossed junction. By dropping gel electrolyte containing 8wt% sorbitol, 33wt% PSS, 0.1 M sodium perchlorate, 12wt% glycol, and deionized H_2O at the junction using micro-syringes, it will form a transistor node. The channel distance of the transistor node can be controlled by the diameter of the spacer cotton wire. Each transistor node can be encapsulated by coating a layer of polymethyl methacrylate (PMMA). The conductive ends of each device node were connected to a data acquisition device of an electrochemical workstation (CHI660D, Chenhua, Shanghai, China) for the electrochemical and electrical test.

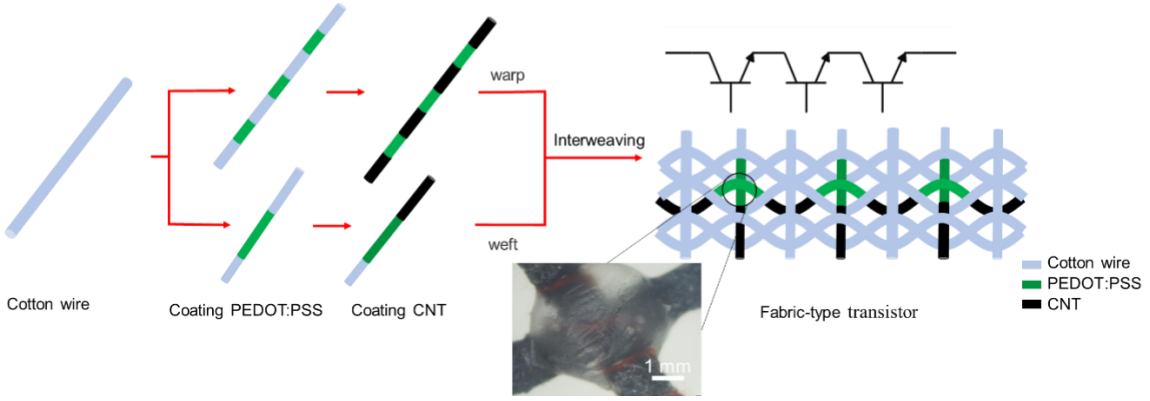

Supplementary Fig. 5 Fabrication process of fabric-type transistors.

For the typical weaving process of a part of fabric-type transistor circuits containing three transistor nodes, two types of cotton/PEDOT: PSS wires were previously fabricated. One was coated with three sections of PEDOT: PSS and four sections of MWCNT by turn along the cotton wire, which can be noted as type-I PEDOT: PSS/cotton wire. The other was only coated with one section of PEDOT: PSS and one section of MWCNT at the proper place of the cotton wire, which can be noted as type-II PEDOT: PSS/cotton wire. Both types of PEDOT: PSS/cotton wires can be interwoven via a shuttle flying processing on an industrial available knitting machine, following different weaving patterns. The stress intension on the PEDOT: PSS/cotton wire was nicely tuned using the self-developed pulley equipment. Either cotton/PEDOT: PSS wire can be selected as warp or weft. Herein, for a typical device weaving process as indicated in Supplementary Fig. 5, type-I PEDOT: PSS/cotton wires were selected

as the warp on the fixed shuttle, and type-II PEDOT: PSS/cotton wires were led by a flying shuttle as the weft. To form the transistors, the PEDOT: PSS sections of both types of wires should be matched with the proper node position. Cotton wires can be also woven together with the cotton/ PEDOT: PSS wires as insulated spacer wires. To weave out different hybridizing patterns, some electrodes can be exchanged with others. The textile edge can be fixed to stabilize the textile. After weaving, gel electrolyte can be dropped at the transistor nodes, and then be encapsulated by coating a layer of PMMA, and the viscosity of the electrolyte is very high, which can be easily coated by dipping into suitable polymer solutions.

Fabrication of the wire-type polymer dielectric capacitors. Polymer dielectric capacitors are a kind of fiber polymer dielectric capacitors with winding structures, which are obtained by simply coating. PVDF/N-methylpyrrolidone solution (2.5 g/L) was repeatedly brush coated on copper-coated cotton wire layer-by-layer and dried in the air on a self-designed coating machine until it reached the wanted thickness. Both ends of the PVDF layer were led out by coaxially coating a layer of MWCNTs, to form two conductive terminals on the cotton wire. The encapsulation of the resistor is achieved by coating PMMA layer.

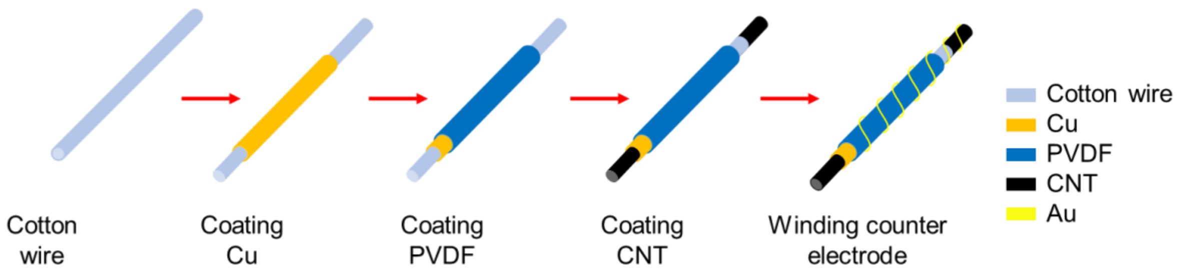

Supplementary Fig. 6 Fabrication process of wire-type polymer dielectric capacitor.

Fabrication of the wire-type resistor. Resistance is fabricated by coating a layer of activated carbon (AC, Kuraray, YP-50f) slurry on the cotton wire, repeatedly, via the self-designed brush coating machine, and then the resistance of different resistance values are obtained according to different lengths. An activated carbon (AC) slurry was made from a 3:1 weight ratio of AC

(Kuraray, YP-50f): PVDF mixture inside the 1-methyl-2-pyrrolidone solution. The encapsulation of the resistor is achieved by coating with a layer of PMMA.

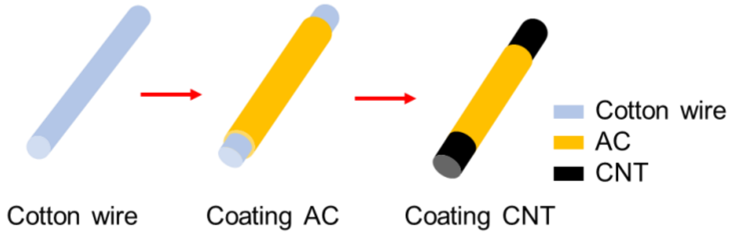

Supplementary Fig. 7 Fabrication process of wire-type resistors.

Fabrication of a fabric-type photovoltaic cell. For the wire-type photoanode of the fabric-type photovoltaic cell, Copper was firstly deposited onto a PBT polymer wire (0.26 mm in diameter). During the chemical plating process, the PBT wire was bathed at 50 °C for 40 min in a solution containing $CuSO_4$ (0.03 M), ethylenediaminetetraacetic acid disodium salt (0.05 M), HCHO (0.1 M), and NaOH (0.2 M). Then, a layer of Mn was electroplated onto the PBT/Cu wire. The PBT/Cu wire was used as the cathode, whereas $PbSb_{0.0003}Sn_{0.0003}Ag_{0.0003}$ alloy rod (diameter = 5.0 mm) was used as the anode. They were kept at a distance of 1 cm and placed in an aqueous electrolyte containing $MnSO_4$ (0.059 M) and H_2SeO_3 (0.27 mM). After electroplating, the polymer/Cu/Mn wire was cleaned by de-ionized water and dried. Then, ZnO-nanowires were deposited on the polymer/Cu/Mn wire by reacting the mixture containing $Zn(CH_3CO_2)_2$ (0.01 M) and hexamethylenetetramine (0.01 M) at 95°C overnight. After being taken out and cleaned with deionized water, the ZnO layer was dried in a vacuum and soaked in the N719/C_2H_5OH solution for one day. Then, the CuI layer was coated by brushing the CuI/CH_3CN solution (nitrogen environment, 130 ^{o}C), which serves as a skin-harmless hole-transmission material.

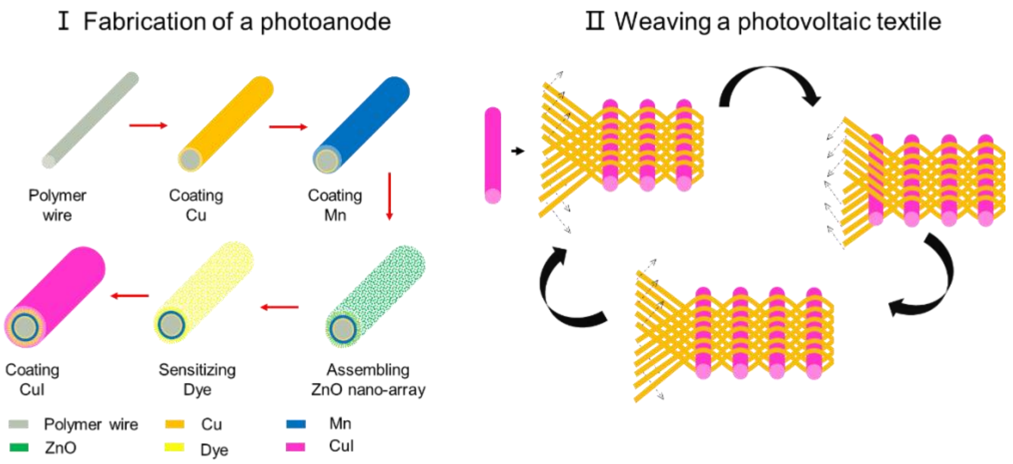

Supplementary Fig. 8 Fabrication process of the wire-type photoanode and fabric-type photovoltaic cell.

Au-coated Cu wire as the counter electrode was interwoven with the CuI coated photoanodes electrode. The two types of electrodes were woven on a modified knitting machine into different weaving patterns. The stress intension along the strings was nicely tuned using the self-developed pulley equipment for effective interaction of photoanode and counter electrodes. The edge of the textile was also stuck firmly for stable operations. The encapsulation of the resultant all-solid photovoltaic textile is achieved by coating a layer of PMMA.

Construction of Zn/MnO_2 Wires. The MnO_2 was synthesized via a hydrothermal process. Firstly, dissolve 43mg $MnSO_4$ and 660 μl H_2SO_4 (9.2 M) in 63 mL water. Secondly, under stirring, slowly add 14 mL aqueous $KMnO_4$ (0.1 M) into the solution and kept stirring for 2 h and then pour it into a hydrothermal reactor. React at 120 °C for 12 h to obtain MnO_2.

To fabricate the MnO_2-coated electrodes, the 2:7:1 weight ratio carbon black (CB), MnO2, and polyvinylidene fluoride (PVDF) mixture in 1-Methyl-2-pyrrolidinone was applied for the production. Then, wire-type MnO_2-coated electrodes were assembled by coating the MnO_2 slurry onto PET/MWCNTs wires.

To fabricate PET/MWCNTs/Zn electrodes, Zn was electrodeposited on PET/MWCNTs wires from electrolytes containing $ZnSO_4$(0.55 M), H_3BO_3(0.32 M), and polyacrylamide(1 g/L). The current was kept at a constant of 30 mA/cm. After electro-deposition, PET/MWCNTs/Zn

electrodes were twisted with thin cotton wires to free them from short-circuiting.

For the final battery assembling, there are two types of electrolytes. The gel electrolyte was prepared by dissolving PVA (10 g) at 90°C into a 100 mL mixture of LiCl (3 M), $ZnCl_2$(2 M), and $MnSO_4$(0.4 M), followed by brush-coating to Zn/MnO_2 electrodes. MnO_2-coating electrodes and PET/MWCNTs/Zn electrodes can be paired and stuck together after the gel electrolyte was cooled down and solidified in the air and then encapsulated in PMMA layers to form battery wires and were winded with cotton wires for better appearance and comfortability. To facilitate the encapsulation process, the electrolyte was solidified by adding water-soluble polymers, such as PVA. After drying in the air, the two-wire electrodes were buried inside the polymer networks with many porous ions channels to hold the electrolyte. As indicated below, the viscosity of the electrolyte is very high, which can be easily coated by dipping into suitable polymer solutions or a melt of thermoplastic polymer. Furthermore, the encapsulated Zn-MnO2 battery was twined inside thin colored cotton wires, which would be further protected from friction. The battery wires can be then woven into textiles as part of either warp or weft, by repeating the shuttle-flying cycle.

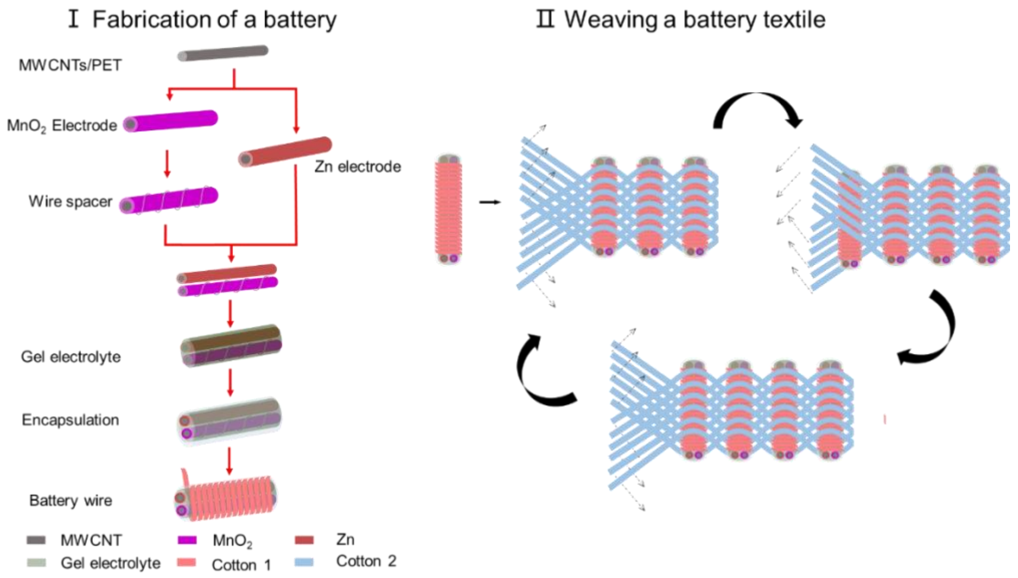

Supplementary Fig. 9 Fabrication process of the wire-type battery and fabric-type battery.

Fabrication of the wire-type infrared light-emission diodes. On a polymer strip of 1mm in

width, two thin Au wires (diameter=0.033 mm) were stuck at a distance of <1 mm. Then, infrared chips (A1GaAs, SLLT6393A, Shenzhen Shenlan Technology Co., Ltd) with two electrode terminals (1.6 mm×0.8 mm×0.6 mm) were aligned in a head-to-tail direction and stuck one-by-one on the back of the polymer strip. Their two electrode terminals were electrically connected to the two Au wires by conductive paste, respectively, in order to achieve the connection in parallel. After sealed by transparent polymer, it can form an infrared light-emission cable. For large-scale fabrication of the light-emission wire, a scalable thermal drawing process can be employed, by using infrared LED chips.

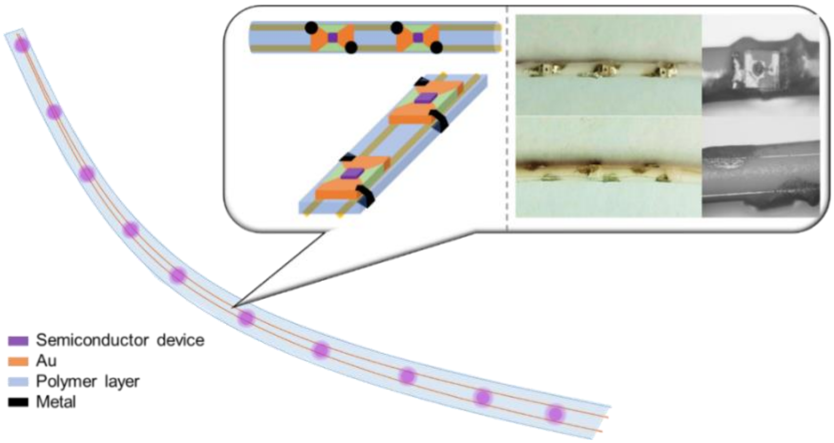

Supplementary Fig. 10 Fabrication process of the wire-type infrared emission module.

Weaving process for the non-printed integrated-circuit textile. Comparing with the industrial weaving process for patterned fabrics, the whole weaving process for the integrated electronic fabric possesses two differences. One difference is that the strings of warp or weft would employ both different colored strings and various function strings. The other difference is that the pattern of the function strings is designed according to the circuit wiring.

For the function strings, not only wire-type electrodes and single-function devices, such as sensor, battery, resistor, and capacitor, were fabricated in advance as discussed above for use as different strings of warp or weft, but also wire-type integrated devices containing two or more devices along one wire was assembled.

A typical wire-type integrated device (denoted as integrated wire device I) containing both

optical sensors integrated with the gate electrode of the transistor was fabricated as indicated in Supplementary Fig. 11. Firstly, a PBT wire coated with a section of Cu layer was successively coated by composited layers of Mn/ZnO/dye/CuI in turn with suitable length. Secondly, a coaxial section of the PEDOT: PSS layer was coated at another position of the PBT layer for fabricating the gate electrode of the transistor. Thirdly, two separated sections of conductive MWCNTs sections were coated as the conductive ends of the gate electrode. Then, Au wire was winded around the Mn/ZnO/dye/CuI layer and extended to the gate electrode to form an electrical connection. After that, it can be interwoven with another PEDOT: PSS wire electrode to form a transistor node.

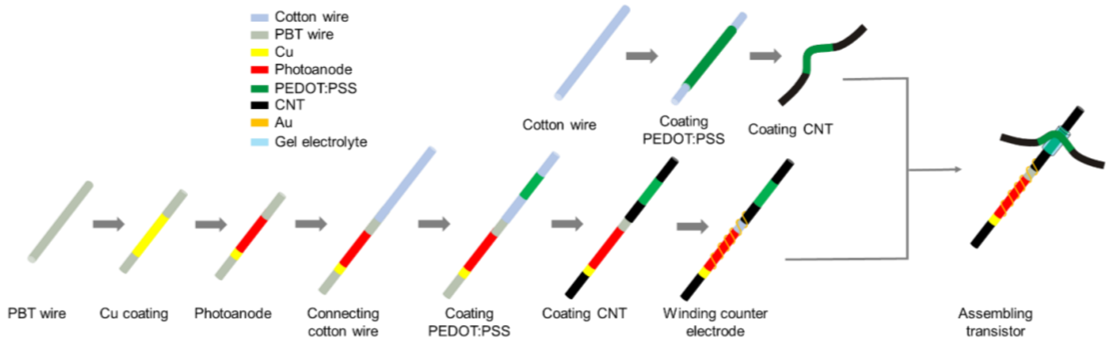

Supplementary Fig. 11 Fabrication process of the integrated wire device I.

Another typical wire-type integrated device (denoted as integrated wire device II) containing both resistor and battery was fabricated as indicated in Supplementary Fig. 12. The integrated wire device II contains two electrodes. One is the Zn electrode, the other the resistor integrated with MnO_2 electrode. For the Zn electrode, a layer of metal Zn was electrodeposited on an MWCNTs -coated cotton wire. For the resistor integrated with the MnO_2 electrode, the cotton wire was firstly coated by a section of AC with suitable length, and secondly coated by two separated conductive MWCNTs sections. Then, the MnO_2 slurry was coated onto one MWCNTs section. After the fabrication of the two electrodes, they were aligned side-by-side by matching the MnO_2 section with the Zn electrode section. The gel electrolytes were then brush-coated onto Zn/MnO_2 electrode pairs, which were then dried in air to stick two electrodes

together. Finally, they were encapsulated in PMMA layers.

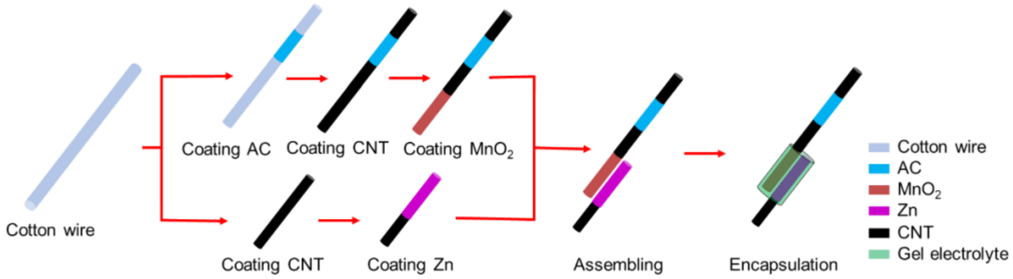

Supplementary Fig. 12 Fabrication process of the integrated wire device II.

During weaving, the circuit wiring of the complete integrated electronic fabric can be fully accomplished along with the shuttle-flying weaving process by simply changing the type of strings for warp and weft, as indicated in Supplementary Fig. 13. Various types of warp strings were prefixed on the fixed shuttle and aligned one-by-one according to our circuit design. During the weaving process, one end of each warp was fixed on a specially designed harness, so that all the warps were tilted up-and-down alternately to form a clamping opening. Then, different weft strings can be fed into the clamping opening. After a weft was fed into the clamping opening, the tilting direction of each warp would alternately change and form a new clamping opening. By repeating the weaving cycle, the circuit wiring process would be extended as designed towards a complete the non-printed integrated-circuit textile.

The electrical connection between different devices can be achieved by either electro-contacting at the crossover node between the MWCNTs coated sections of both warp and weft, or along one wire by coated conductive MWCNTs sections. After weaving, all the interwoven device nodes were encapsulated by PMMA as a whole. In a word, the pattern weaving process may look largely different from the existed circuit printing process, but very similar to the industrial textile making process.

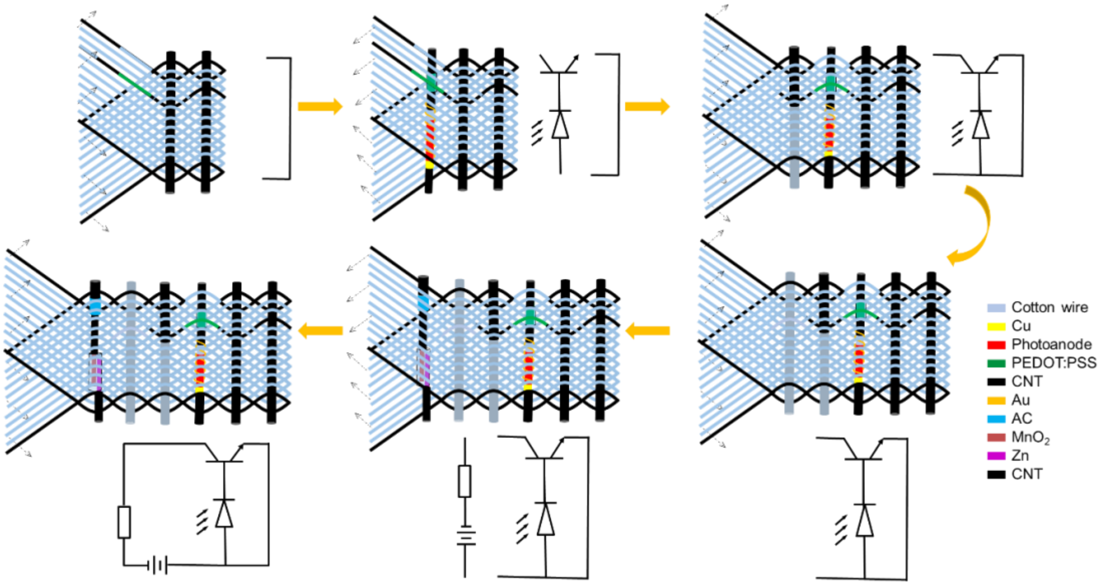

Supplementary Fig. 13 Typical weaving process for the circuit wiring of a part of a non-printed integrated-circuit textile.

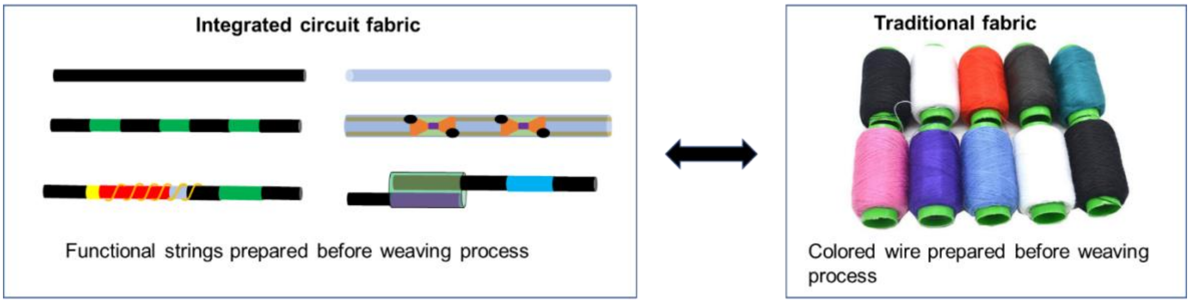

Supplementary Fig. 14 The functional strings for the non-printed integrated-circuit textile can serve similarly as colored wires for traditional fabrics during the weaving process.

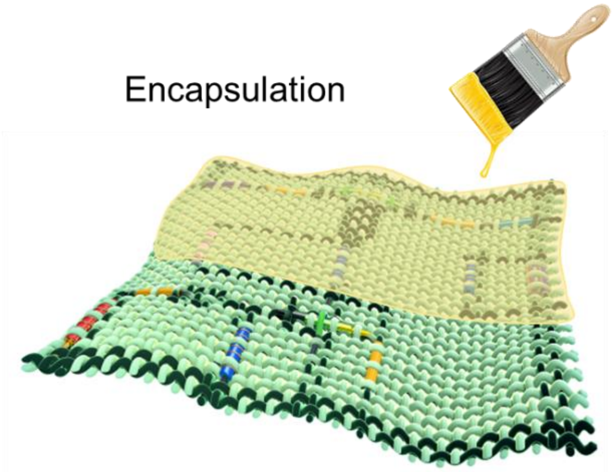

Supplementary Fig. 15 Encapsulation process for the non-printed integrated-circuit textile. The all-solid NIT fabric could be easily encapsulated via a dry heat-wrapping process.

Supplementary Note 2: The textile non-printed integrated circuit, is different from existing printed electronics on soft plastics.

Many works on flexible electronics are based on soft plastics, which are fabricated by printing semiconductive function materials layer by layer, and typically appear on the human body as mosaic, tattoo, or bandages. With also concerns on biocompatibility and suitability, printed electronics on soft plastics have made progress in implementing functions of sensing or wireless communication in a bendable or stretchable way.

As a feature of all human societies, the habit of wearing fabric can be dated back to prehistoric times. By covering the human body surface, fabrics are exposed to a vast trove of optical, mechanical, chemical, and biological information from the daily environment, which could form a massive dataset, and make sense for human health. Clothing industry giants are also pursuing a dream of “fabric computer” or “smart assistant hidden in the clothes”, by exploring a better way of merging electronics with common textile. A lot of efforts were made to develop fiber-shaped devices, such as fibrous solar cells, fibrous batteries, fibrous sensors, and fibrous LEDs, etc. Herein, we have for the first time stepped from single-function fibrous devices to an integrated textile system, which can accomplish a complete set of tasks, including signal transduction, conditioning, logic judgment, and wireless data transmission. The fabrication of the entire system has directly taken a different strategy of textile weaving, rather than printing on fabrics or soft plastics. With almost the same appearance as everyday wearing fabrics, they hold a better appeal to the wearer who is at his or her optimal health.

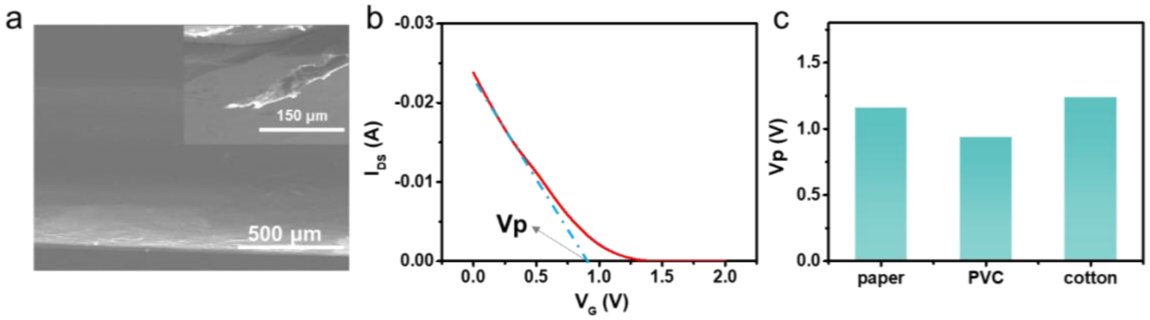

Supplementary Fig. 16 The performance of transistors. **a** SEM images for the PEDOT: PSS electrode (Insert: sectional view). **b** The pinch-off voltage (V_P) is calculated by the intercept of the line extrapolated. **c** The V_P values of transistors fabricated on different substrates.

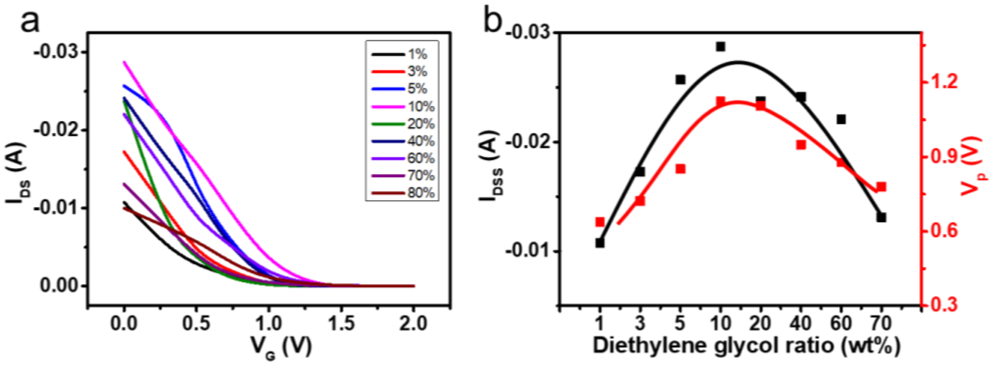

Supplementary Fig. 17 Performance of transistor with different diethylene glycol ratio in the PEDOT: PSS layer. **a** The transfer characteristic curves of transistors for different diethylene glycol ratios. **b** The saturated drain current (I_{DSS}) and V_p value of transistor for different diethylene glycol ratio.

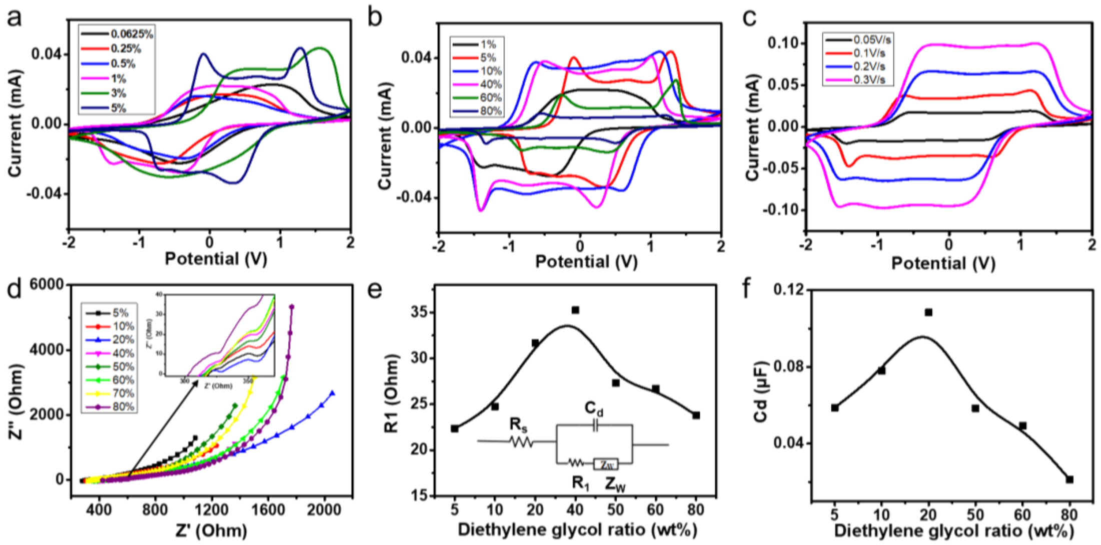

Supplementary Fig. 18 Electrochemical tests of transistors with different diethylene glycol ratios in the PEDOT: PSS layer. **a, b** The cyclic voltammetry curves for different diethylene glycol ratio. **c** The cyclic voltammetry curve at different scan rates. **d** Nyquist plots of transistors with different diethylene glycol ratio in the PEDOT: PSS layer. Electrochemical impedance spectroscopy (EIS) measurement was performed at 0V, 0.10 Hz – 100 kHz, with an AC voltage amplitude of 5 mV. **e** The value of charge-transfer resistance (R_1) at different diethylene glycol ratio. **f** The value of double-layer capacitance (C_d) at different diethylene glycol ratio.

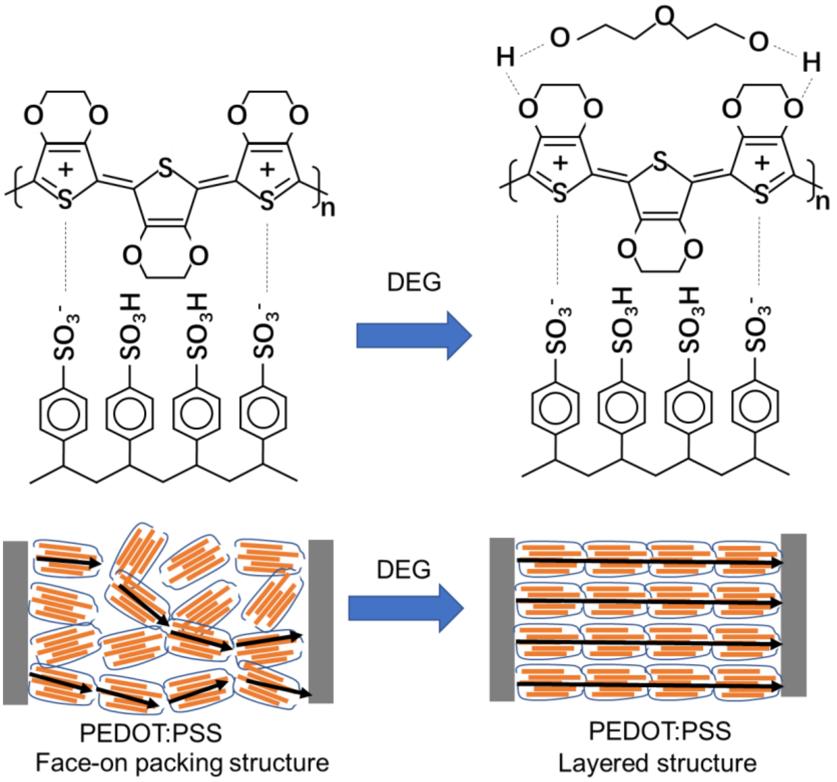

Supplementary Fig. 19 Morphological change in the PEDOT:PSS films caused by the addition of DEG.

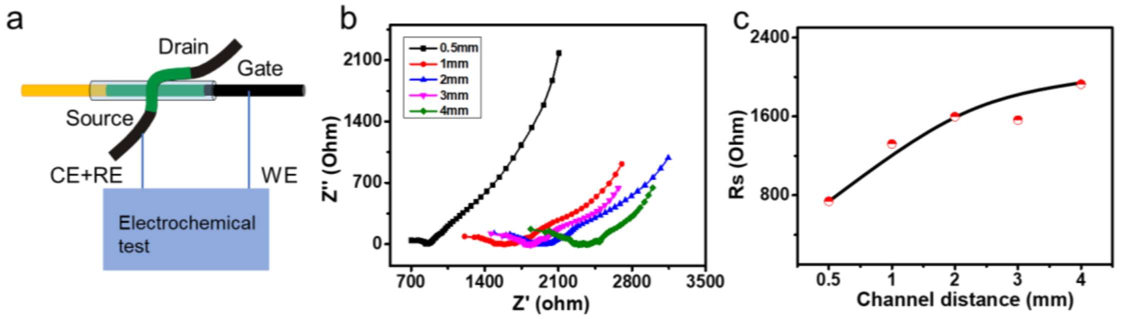

Supplementary Fig. 20 Electrochemical tests of transistor (10 wt% of diethylene glycol and 0.5wt% of PEG-2000) at different channel distances. **a** Schematic diagram of the electrochemical test. **b** Nyquist plots for different channel distance. **c** The value of total resistance (R_S) for different channel distance.

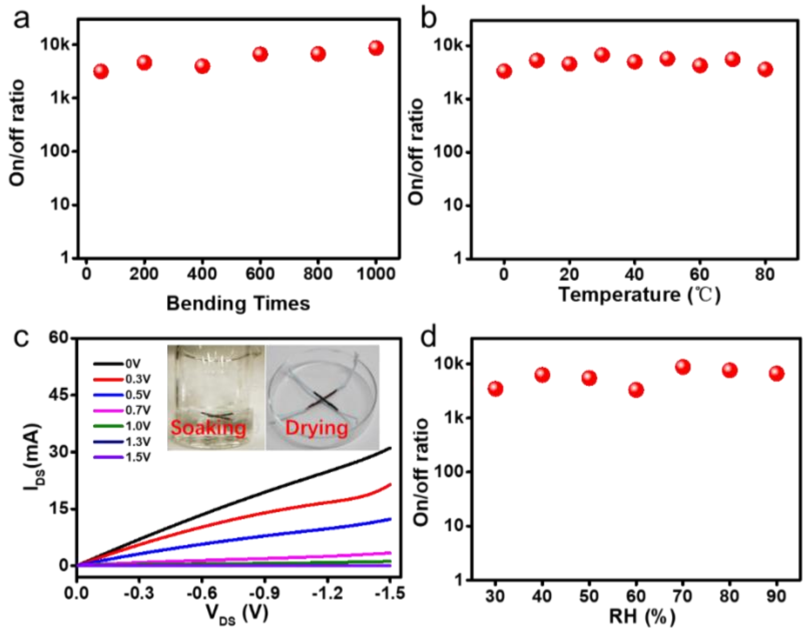

Supplementary Fig. 21 The stability of bending, temperatures, waterproof, and humidity after encapsulation. **a** The on/off ratio of the transistor at different bending times. **b** The on/off ratio of the transistor at different temperatures. **c** The transistor is waterproof. **d** The on/off ratio performance of the transistor at different humidity.

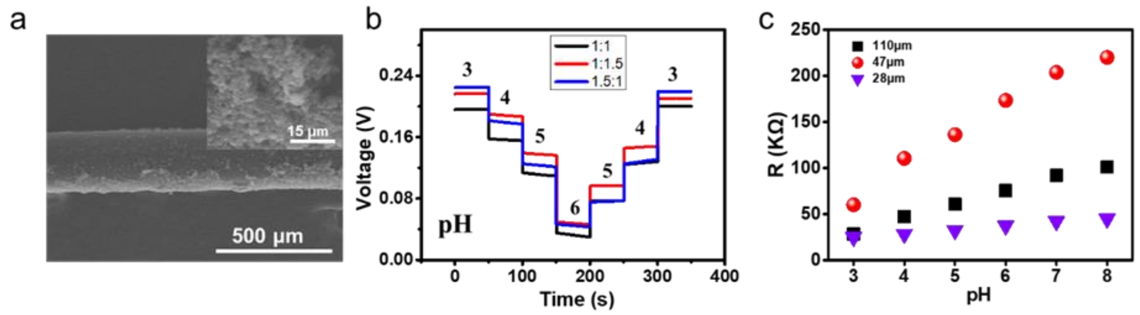

Supplementary Fig. 22 The performance of wire-type pH sensor. **a** SEM images for a PANI electrode (Insert: sectional view). **b** The sensitivity of pH sensor based on PANI synthesized at different molar ratios of $(NH_4)_2S_2O_8$ and aniline. (During the test of sweat sensor, the interval time between different pH values is 6s.) **c** The performance of the pH sensor on different thicknesses of polyaniline.

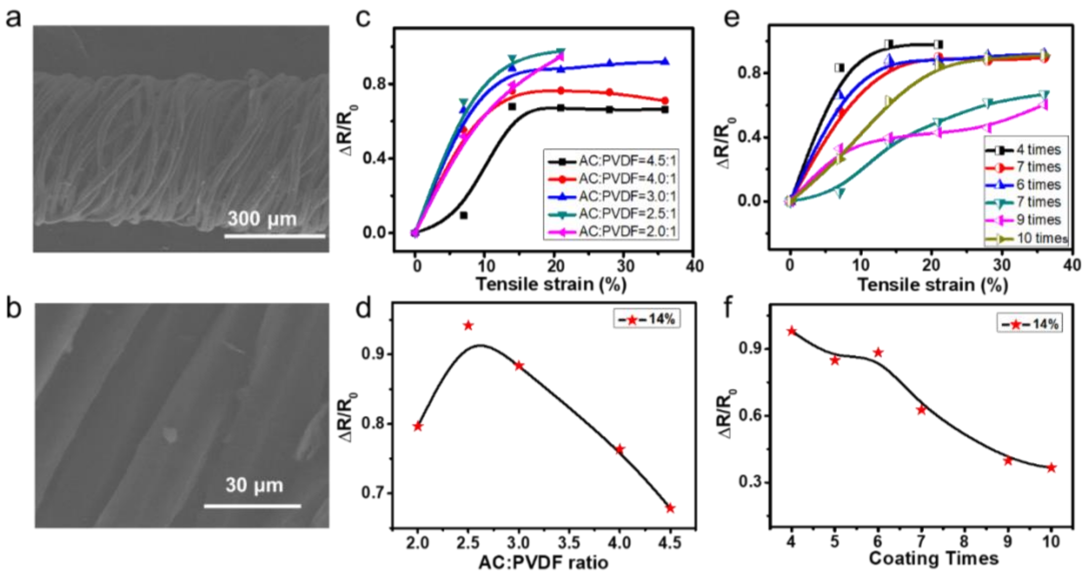

Supplementary Fig. 23 The performance of wire-type tensile stress sensor. **a, b** SEM images for the wire-type tensile stress sensor. **c** Optimization on the different concentrations of AC. **d** The $\Delta R/R_0$ value of different AC concentrations based on 14% tensile strain. **e** Optimization on the number of dip-coating cycles. **f** The $\Delta R/R_0$ value of dip-coating cycles based on 14% tensile strain.

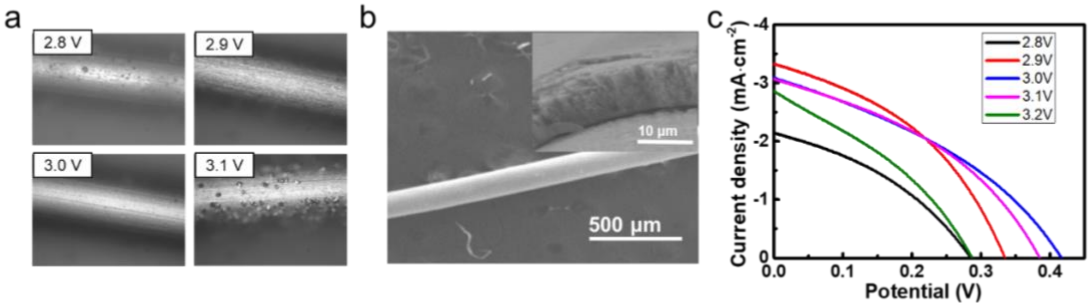

Supplementary Fig. 24 The performance of wire-type optical sensor. SEM images for: **a** the metal electrode of different metal plating voltage conditions; **b** the wire-type photoanode of the optical sensor (Insert: sectional view). **c** Electric output performance of the wire-type optical sensor with different manganese plating voltage.

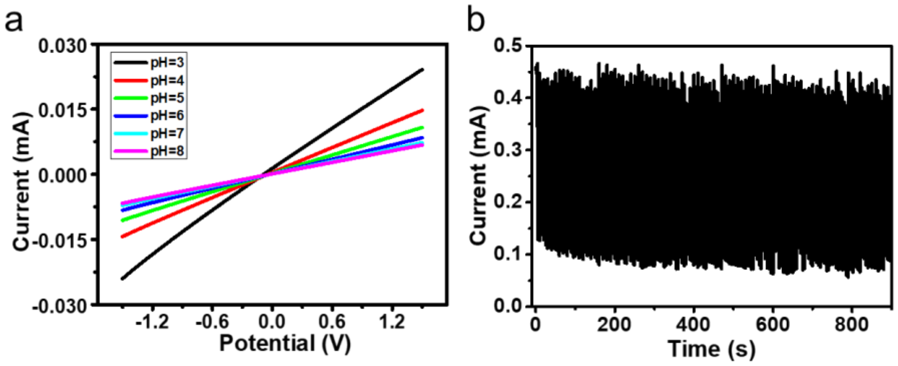

Supplementary Fig. 25 The PANI was synthesized from $(NH_4)_2S_2O_8$: aniline with a molar ratio of 1:1. **a** The current-voltage curves of wire-type pH sensor at different pH values. **b** Durability test of the pH sensor.

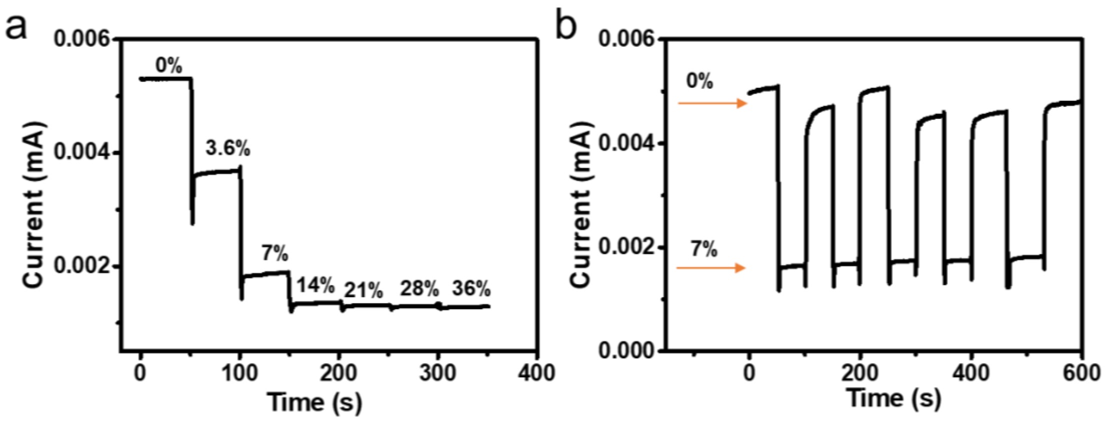

Supplementary Fig. 26 The performance of wire-type tensile stress sensor: **a** sensitivity and **b** output stability of tensile stress sensor.

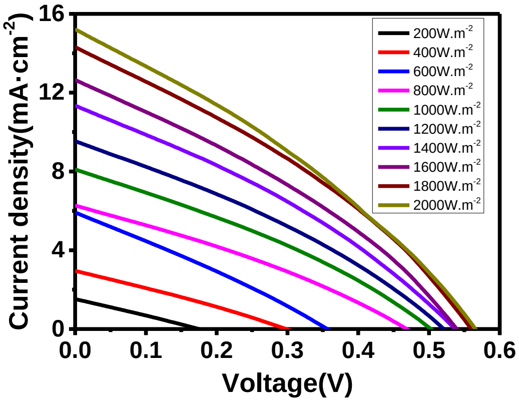

Supplementary Fig. 27 The current density-voltage curves of wire-type optical sensor at different light intensity. The device fabrication conditions are as follows: a layer of ZnO-nanowire arrays was grown on the Mn plated substrate in a solution of zinc acetate (0.03 M) and hexamethylene tetramine (0.03 M) at 95°C overnight. After cleaning with deionized water and drying in a vacuum, the as-prepared photoanode was sensitized in an ethanol solution of N719 for 24 h. After that, Au/Cu wire was twisted around the CuI coated wire electrode and sealed in polymethyl methacrylate (PMMA) to form the wire-type optical sensor.

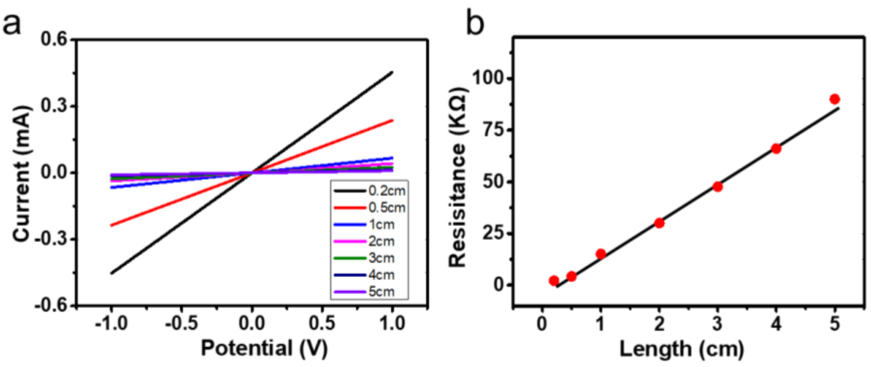

Supplementary Fig. 28 Optimization of the wire-type resistance on the axial-direction of the layer by coating 3:1 AC/PVDF. **a** Current-voltage performance. **b** dependence of the resistance value on the axial-direction length.

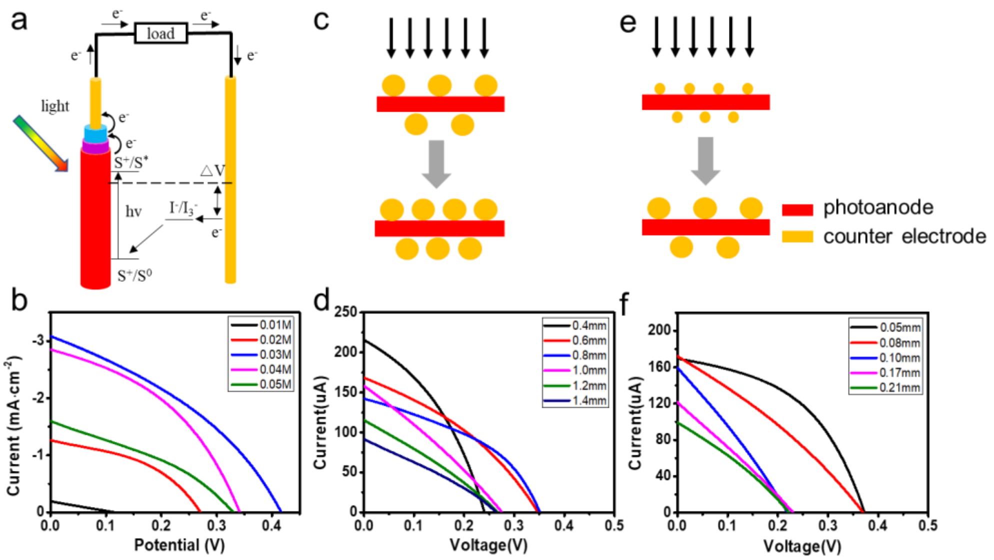

Supplementary Fig. 29 Electrical output of the photovoltaic textile. **a** The basic working principle. **b** Optimization of the [Zn^{2+}] condition during fabrication for power output performance. **c** Schematic illustrations of photovoltaic textiles with different interval distances of counter electrodes. **d** Photovoltaic textile performance at various interval distances with [Zn^{2+}]=0.03M photovoltaic devices. **e** Scheme of photovoltaic textiles with different diameters of counter electrodes. **f** Performance dependence on the diameter of counter electrodes with [Zn^{2+}]=0.03M photovoltaic devices.

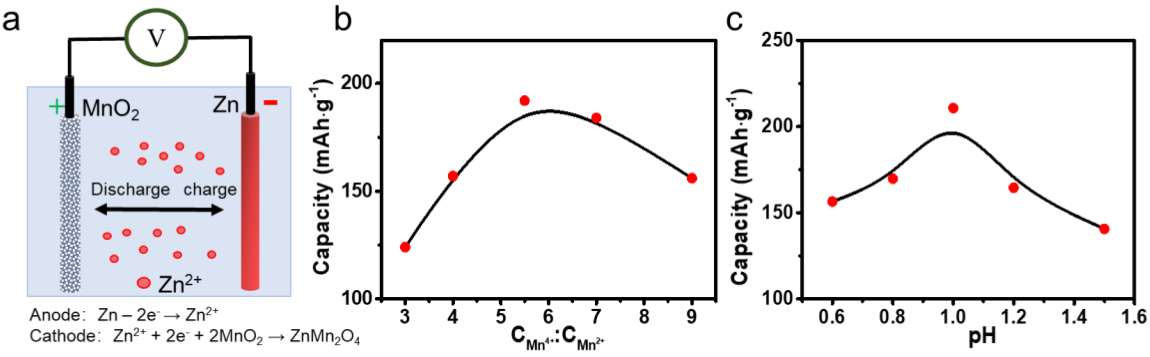

Supplementary Fig. 30 Optimization of Zn-MnO_2 battery wires. **a** The working principle of Zn-MnO_2 battery wire. Capacity during the initial charge-discharge cycle at 0.5 C for MnO_2 electrode fabricated at: **b** different concentrations of Mn(VII)/Mn(II) ratios and **c** pH condition.

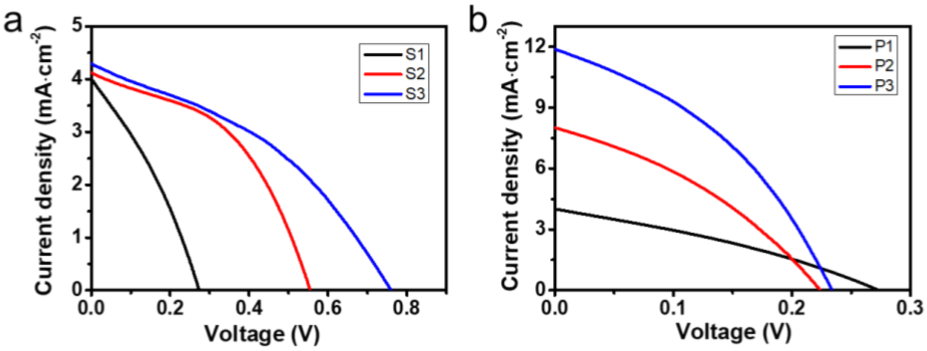

Supplementary Fig. 31 Power output of the photovoltaic textiles for different electrical connection: **a** in series, **b** in parallel.

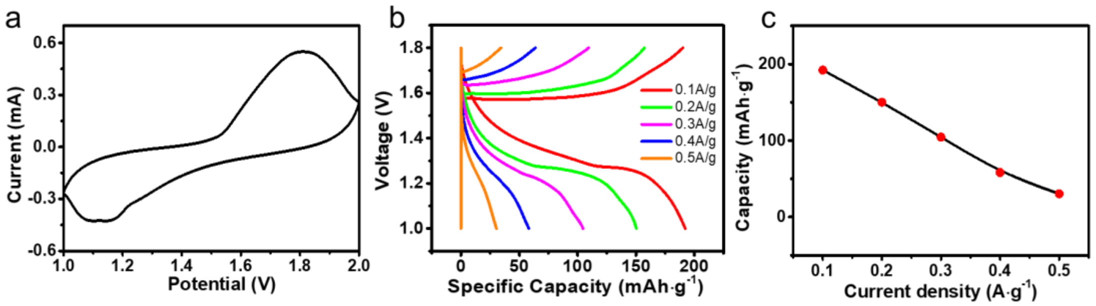

Supplementary Fig. 32 Zn/MnO_2 battery performance. **a** Cyclic voltammetry curves at different scanning rates. **b** Charge-discharge curves. **c** Capacity curves at different discharge currents.

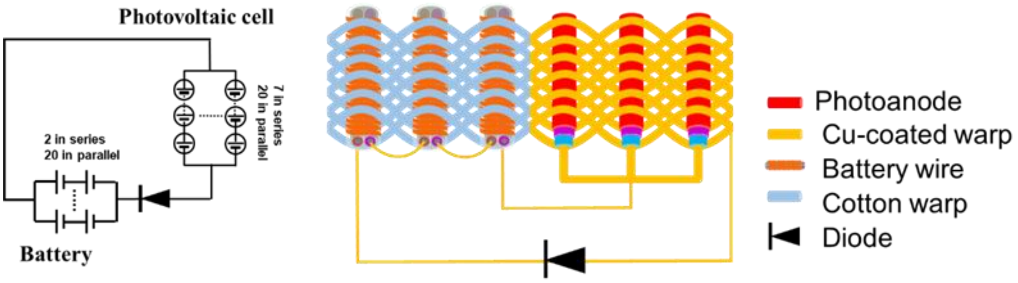

Supplementary Fig. 33 Schematic illustration of photo-charging power module with blocking diodes.

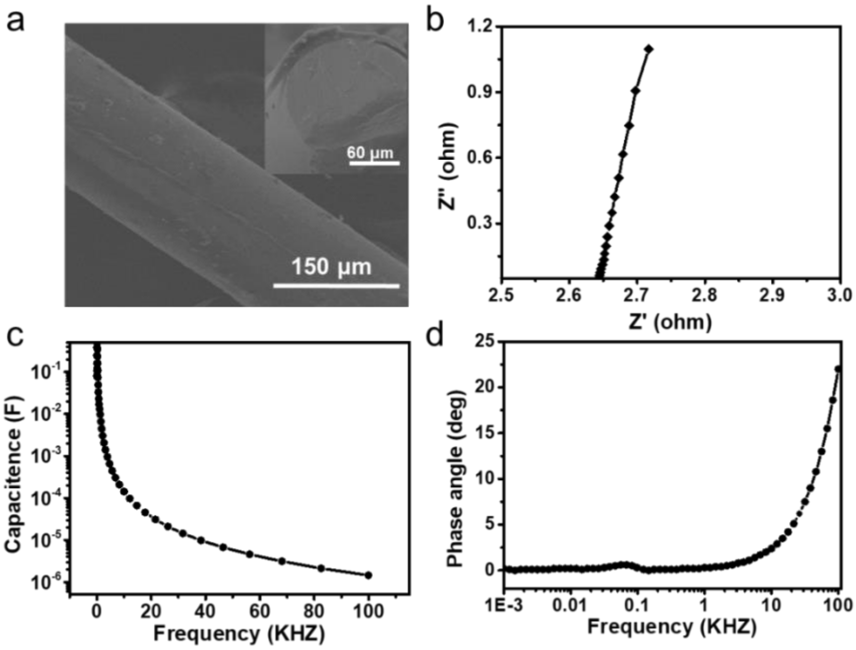

Supplementary Fig. 34 Performance of wire-type polymer dielectric capacitors. **a** SEM images for PVDF electrode (Insert: sectional view). **b** Nyquist diagram of polymer dielectric capacitors. **c** Plot of capacitance versus frequency. **d** Plot of impedance phase angle versus frequency.

Supplementary Table 1. Logic response table with 3 levels of light-emission intensity

Condition			Light-emission level	Alarm type
Light	Body moving	Sweating		
N	N	N	0	No alarm
N	Y	N	2	Sound and light
N	N	Y	2	Sound and light
N	Y	Y	2	Sound and light
Y	N	N	0	No alarm
Y	Y	N	1	Sound
Y	N	Y	1	Sound
Y	Y	Y	1	Sound

N: no
Y: yes

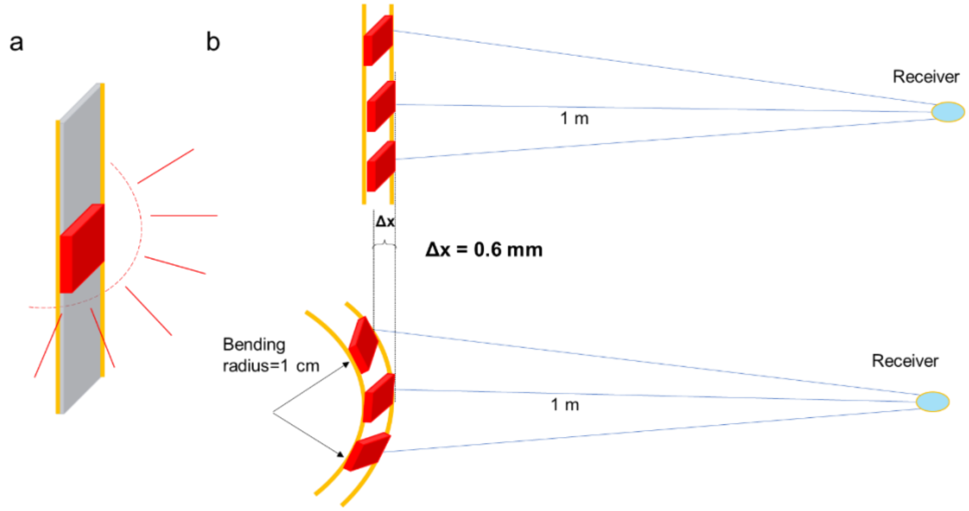

Supplementary Fig. 35 The emission direction of infrared emission fiber. **a** Schematic diagram of emission direction of an infrared emission fiber. **b** The bending has little influence on the detection of infrared emission for practical wearable applications.

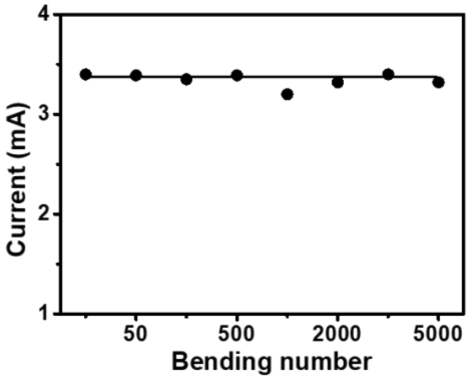

Supplementary Fig. 36 The bending stability of the infrared light emission.

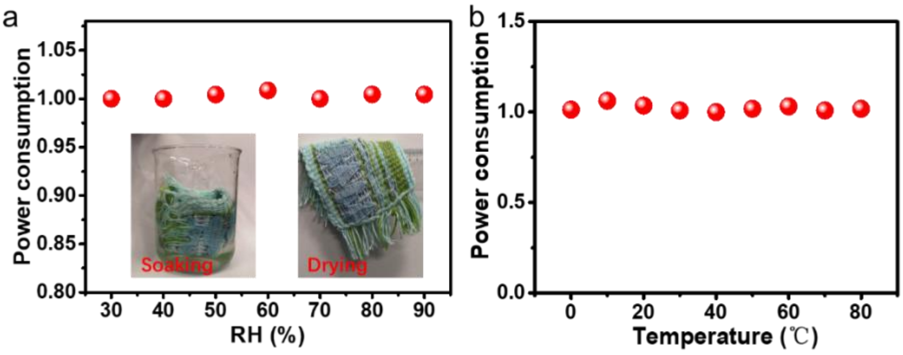

Supplementary Fig. 37 a The power consumption of NIT fabric under different humidity. **b** The power consumption of NIT fabric under different temperatures.
