# Supplementary material for: A non-printed integrated-circuit textile for wireless theranostics
Source: Nat Commun. 2021 Aug 12;12:4876. doi: 10.1038/s41467-021-25075-8 (PMC8361012; doi:10.1038/s41467-021-25075-8)
Supplement: Supplementary file 4 — Description of Additional Supplementary Files [file 41467_2021_25075_MOESM4_ESM.pdf]

Title: Supplementary Movie 1:
Description: Wireless transmission to mobile terminal by an integrated circuit fabric

Title: Supplementary Movie 2:
Description: All-woven integrated circuit fabric choose different alarms for different stimulations
